# Transcriptome Analysis of Dnmt3l Knock-Out Mice Derived Multipotent Mesenchymal Stem/Stromal Cells During Osteogenic Differentiation

**DOI:** 10.3389/fcell.2021.615098

**Published:** 2021-02-25

**Authors:** Chih-Yi Yang, Rita Jui-Hsien Lu, Ming-Kang Lee, Felix Shih-Hsian Hsiao, Ya-Ping Yen, Chun-Chun Cheng, Pu-Sheng Hsu, Yi-Tzang Tsai, Shih-Kuo Chen, I-Hsuan Liu, Pao-Yang Chen, Shau-Ping Lin

**Affiliations:** ^1^Institute of Biotechnology, National Taiwan University, Taipei, Taiwan; ^2^Institute of Plant and Microbial Biology, Academia Sinica, Taipei, Taiwan; ^3^Department of Medicine, Washington University in St. Louis, St. Louis, MO, United States; ^4^Department of Animal Science and Biotechnology, Tunghai University, Taichung, Taiwan; ^5^Institute of Molecular Biology, Academia Sinica, Taipei, Taiwan; ^6^Department of Life Sciences, National Taiwan University, Taipei, Taiwan; ^7^Department of Animal Science and Technology, National Taiwan University, Taipei, Taiwan; ^8^Agricultural Biotechnology Research Center, Academia Sinica, Taipei, Taiwan; ^9^Center for Systems Biology, National Taiwan University, Taipei, Taiwan; ^10^Research Center for Developmental Biology and Regenerative Medicine, National Taiwan University, Taipei, Taiwan

**Keywords:** epigenetics, DNMT3L, DNA methylation, bone-marrow MSCs, osteogenesis

## Abstract

Multipotent mesenchymal stem/stromal cells (MSCs) exhibit great potential for cell-based therapy. Proper epigenomic signatures in MSCs are important for the maintenance and the subsequent differentiation potential. The DNA methyltransferase 3-like (DNMT3L) that was mainly expressed in the embryonic stem (ES) cells and the developing germ cells plays an important role in shaping the epigenetic landscape. Here, we report the reduced colony forming ability and impaired *in vitro* osteogenesis in *Dnmt3l*-knockout-mice-derived MSCs (*Dnmt3l* KO MSCs). By comparing the transcriptome between undifferentiated *Dnmt3l* KO MSCs and the MSCs from the wild-type littermates, some of the differentially regulated genes (DEGs) were found to be associated with bone-morphology-related phenotypes. On the third day of osteogenic induction, differentiating *Dnmt3l* KO MSCs were enriched for genes associated with nucleosome structure, peptide binding and extracellular matrix modulation. Differentially expressed transposable elements in many subfamilies reflected the change of corresponding regional epigenomic signatures. Interestingly, DNMT3L protein is not expressed in cultured MSCs. Therefore, the observed defects in *Dnmt3l* KO MSCs are unlikely a direct effect from missing DNMT3L in this cell type; instead, we hypothesized them as an outcome of the pre-deposited epigenetic signatures from the DNMT3L-expressing progenitors. We observed that 24 out of the 107 upregulated DEGs in *Dnmt3l* KO MSCs were hypermethylated in their gene bodies of DNMT3L knock-down ES cells. Among these 24 genes, some were associated with skeletal development or homeostasis. However, we did not observe reduced bone development, or reduced bone density through aging *in vivo*. The stronger phenotype *in vitro* suggested the involvement of potential spreading and amplification of the pre-deposited epigenetic defects over passages, and the contribution of oxidative stress during *in vitro* culture. We demonstrated that transient deficiency of epigenetic co-factor in ES cells or progenitor cells caused compromised property in differentiating cells much later. In order to facilitate safer practice in cell-based therapy, we suggest more in-depth examination shall be implemented for cells before transplantation, even on the epigenetic level, to avoid long-term risk afterward.

## Introduction

Stem cells that are capable of self-renewal and differentiation, play very important roles to maintain dynamic but sustainable tissue homeostasis (Collins et al., 2005). This delicate balance relies on cooperative interactions between transcription networks and epigenetic regulations, including DNA methylation, histone modifications and non-coding RNA-mediated modulation of gene expression (Reik, 2007; Lunyak and Rosenfeld, 2008; Bloushtain-Qimron et al., 2009; Atlasi and Stunnenberg, 2017). Multipotent mesenchymal stem/stromal cells (MSCs) have a trilineage differentiation ability toward osteoblasts, chondrocytes and adipocytes (Pittenger et al., 1999). In accordance with their ability to differentiate into multiple lineages, and having immunomodulatory function(s), MSCs can be isolated from various origins, such as bone marrow, adipose tissues (Zuk et al., 2002), umbilical cord blood (Erices et al., 2000), skeletal muscle (Young et al., 2001), amniotic fluid (In t Anker et al., 2003), placenta (Miao et al., 2006), and dental pulp (Lin et al., 2011; Berebichez-Fridman and Montero-Olvera, 2018; Spagnuolo et al., 2018). With the long history of successful MSC-containing bone marrow transplantations, as well as the availability from other accessible origins like tooth, placenta, umbilical cord blood, amniotic fluid and adipose tissues, MSCs are one of the top candidate cell types available for allogenic and autologous cell therapy in regenerative medicine (Ballini et al., 2017; Han et al., 2019). The therapeutic potential of MSCs is mainly based on their ability to modulate immune response (Abdi et al., 2008; Gao et al., 2016). The strong anti-inflammatory effect and paracrine activity from their secretory proteins and functional RNAs can be delivered into remote organs or tissues via extracellular vesicles (Chen et al., 2019). Preclinical and clinical trials on MSCs based therapy have been carried out in many countries, in order to improve the treatment for the injured bone, cartilage, and the autoimmune diseases.

However, one of the limitations of using MSCs in regenerative medical applications is their gradual loss of multipotency and therapeutic properties under culture conditions. MSCs cultured for prolonged periods or isolated from elder individual are less efficient in their trilineage differentiation ability, with the impairment in osteogenesis being particularly significant (Digirolamo et al., 1999; Bonab et al., 2006; Zhou et al., 2008; Yang, 2018). Optimization of culture conditions can sustain MSC properties better *in vitro* (Sotiropoulou et al., 2006), and make them safer and more suitable for clinical applications. These include the introduction of better culture surface (Engler et al., 2006; Lee et al., 2017), hypoxia condition (Wang et al., 2020), providing scaffold and other biomaterials (Meinel et al., 2004; Marrelli et al., 2016), to maintain better multipotency or differentiation outcome for the cultured MSCs. In addition, the replacement of FBS by chemically defined or standardized supplements (Bieback et al., 2009; Marrazzo et al., 2016) can facilitate the clinical-grade production of MSCs.

While the culture condition can be optimized to certain extent, the intrinsic defects from the isolated MSCs cannot be easily fixed. Here we report an unexpected observation of compromised osteogenesis differentiation ability of MSCs isolated from DNMT3L deficient mutant mice. DNMT3L is a germ and ES cell enriched epigenetic cofactor (Bourc’his et al., 2001; Hata et al., 2002; Liao et al., 2014). We and others have demonstrated that DNMT3L maintains the quiescence of spermatogonial progenitor cells and prevents exhaustion of stem cell populations to maintain male germ line homeostasis (Bourc’his and Bestor, 2004; Liao et al., 2014). Dnmt3l knock-out mice are infertile (Bourc’his and Bestor, 2004; Webster et al., 2005; Hata et al., 2006), but otherwise develop normally into adulthood without reported somatic phenotypes. DNMT3L does not have enzymatic activity but interacts with DNMT3A and DNMT3B to facilitate *de novo* DNA methylation and thus influences gene expression (Chedin et al., 2002; Guenatri et al., 2013). DNMT3L binds to histone H3 tails in a H3K4methylation sensitive manner, and recruits other histone modifiers through its PHD domain (Aapola et al., 2000; Ooi et al., 2007; Otani et al., 2009; Hashimoto et al., 2010; Zhang et al., 2010). We further demonstrated that ectopic DNMT3L expression can promote the assembly of the HDAC1/TRIM28/SETDB1/DNMT3A/DNMT3L complex and repress transcription of newly infected retroviral sequence independent of DNA methylation (Kao et al., 2014). There has been very limited description of potential DNMT3L functions beyond germ lines and ES cells, partly due to the difficulties in demonstrating its expression in specific progenitor cell types in somatic lineages.

Recently we demonstrated that transient expression of ectopic DNMT3L in later passaged MEFs were sufficient to cause long term epigenomic landscape changes and halt senescence progression (Yu et al., 2020). The transiently expressed DNMT3L facilitated the short-term formation of DNMT3L-DNMT3A-KAP1-SETDB-HDAC1 complex as well as guiding them to certain endogenous retroviruses and retrotransposons to introduce H3K9me3 and reduce histone acetylation in aging fibroblasts (Kao et al., 2014; Yu et al., 2020). DNMT3L also interacted with polycomb group members to facilitate repressive H3K27me3 modifications on certain aging associated derepressed genes. The long-term repressive histone modifications and dramatically prolonged cell proliferation still maintained long after the ectopic DNMT3L is silenced (Yu et al., 2020). In the current study, we tackled a potential long-term effect of transient endogenous DNMT3L expression in ES cells and progenitor cells, associated with cellular property changes of the *Dnmt3l* KO mice derived MSCs (*Dnmt3l* KO MSCs) under culture condition.

We compared the *in vitro* CFU-F formation rate, proliferation and differentiation abilities between MSCs derived from *Dnmt3l* KO mice and their WT littermates. Significantly reduced CFU-Fs and impaired osteogenic ability were observed in Dnmt3l KO mice derived BM-MSCs as well as in another epiphysis-derived MSCs (EMSCs) population (Cheng et al., 2012). The molecular mechanisms associated with this impaired osteogenic ability were investigated by comparing and contrasting the transcriptomes of undifferentiated and differentiating MSCs derived from *Dnmt3l* KO mice and their WT littermates. The injury repair ability of the endogenous MSCs between wild type and *Dnmt3l* KO mice were also compared. As the canonical DNMT3L protein was not detectable in cultured MSCs, various hypotheses were proposed and tested to determine how transiently expressed DNMT3L in ES cells or subsequent progenitor cells contribute to the long-term effect on the subsequent differentiating cell lineages, and their adaptation toward *in vitro* culture conditions.

## Results

### Multipotent MSCs Derived From *Dnmt3l* KO Mice Have Reduced Colony Forming Capacity and Osteogenic Potential Suggesting Compromised Potency *in vitro*

**FIGURE 1:**
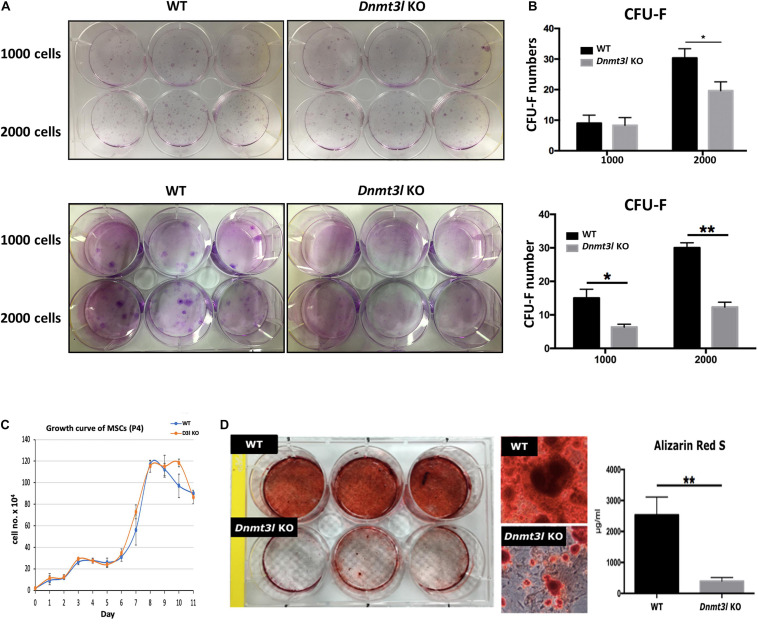
*Dnmt3l* KO BM-MSCs have significantly reduced CFU-F forming rate and impaired osteogenic potential. **(A)** *Dnmt3l* KO and wild type MSCs at passage 4 were seeded at 1000 cells per well or 2000 cells per well density on 6-well plate, and calculate the colony forming unit-fibroblasts (CFU-F) number after 9 days of culture. Two batches of independent studies with mixed BM-MSCs from three mice of each genotype were shown. **(B)** The bar chart shows CFU-F from WT and Dnmt3l KO mice. The CFU-F with diameter greater than 2 mm were considered valid. **(C)** MSCs expanded from passage 3 to 4 were seeded at a density of 2 × 10^4^ cells per well in 12 well plates. Cells from three wells of each genotype were isolated each day for 11 days and were calculated for live cell numbers based on trypan blue dye exclusion. **(D)** MSCs expanded from passage 3 to 4 were seeded at 5 × 10^4^ cells per cm^2^ for each well in 6-well plates. Cells were cultured in osteogenic induction medium once confluence was reached. After 14 days of osteogenic induction, calcium precipitation was quantified by alizarin red S staining. The osteogenic ability was dramatically decreased in MSCs derived from *Dnmt3l* KO mice compared to that in MSCs obtained from their wild-type littermates. The data are presented as the means ± SEM. *p < 0.05, **p < 0.01.

Multipotent MSCs are capable of self-renewal and can differentiate into multiple lineages *in vitro*. We compared and contrasted BM-MSC properties between *Dnmt3l* KO mice and their WT littermate. We did not detect differences in the frequency of MSC cell surface markers tested between genotypes (Supplementary Figure 1). A significantly reduced CFU-F was observed from BM-MSCs and EMSCs (another subtype of MSCs derived from epiphysis from *Dnmt3l* KO mice than those obtained from WT littermates (Figures 1A,B and Supplementary Figure 2A). To clarify whether *Dnmt3l* KO MSCs and EMSCs have an overall lower proliferation activity, or just proliferate poorly in low density, we checked the proliferation curves of BM-MSCs and EMSCs from paired *Dnmt3l* KO mice and wild type littermates. We did not observe major proliferation differences from paired BM-MSCs at passage 4 (Figure 1C), where we used for the differentiation assay. On the other hand, at passage 6, *Dnmt3l* KO EMSCs had reduced proliferation activity compared to those from wild type animals (Supplementary Figure 2B). This is consistent with our previous observations of premature senescence phenotype in cultured *Dnmt3l* KO MEFs (Liao et al., 2015), as well as slightly shorter lifespan for *Dnmt3l* KO compared to wild type littermates (data not shown).

In addition to self-renewal property, MSCs are able to differentiate into osteoblasts, chondrocytes and adipocytes *in vitro* upon induction (Caplan, 1991). We quantified calcium precipitation by ARS staining after 14 days of osteogenic induction and showed that *Dnmt3l-*KO-mice-bone-marrow-derived MSCs (*Dnmt3l* KO MSCs), and *Dnmt3l* KO EMSCs dramatically lost their osteogenic ability (Figure 1D and Supplementary Figure 2C). The compromised osteogenic abilities of *Dnmt3l* KO BM-MSCs could not be attributed merely to reduced proliferation activity of cells in the passage used for differentiation analysis (Figure 1C), which is also indirectly supported by the reasonable adipogenic ability observed on these cells (Supplementary Figure 3). It is reported that aging MSCs favor adipogenic differentiation over osteogenic differentiation (Infante and Rodriguez, 2018). The severely compromised osteogenesis and relatively mild adipogenesis defects are reminiscent of a trend for premature aging event.

### Lack of Canonical DNMT3L Expression in MSCs

**FIGURE 2:**
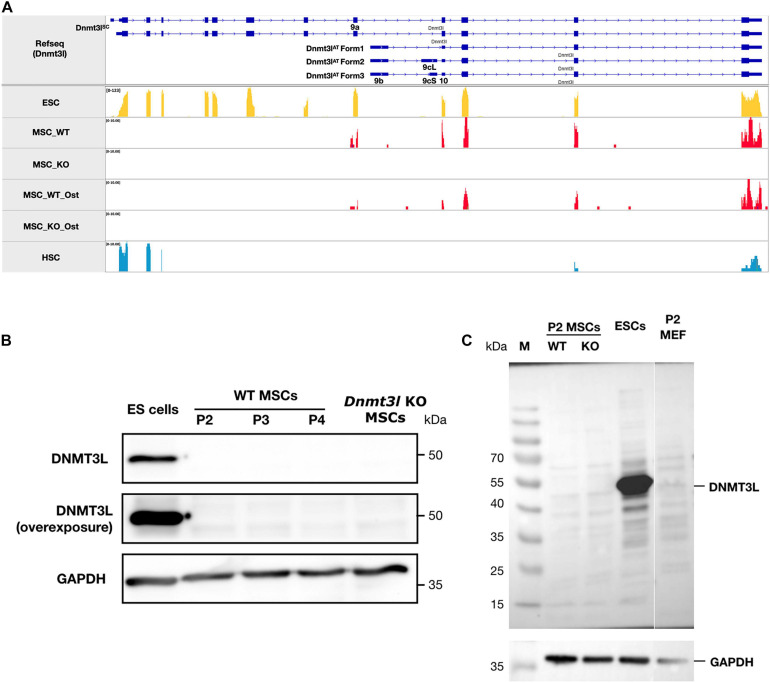
The ∼50 kDa canonical stem cell form of DNMT3L protein is not detectable in MSCs. **(A)** No sequencing reads were mapped to exons 1–8 of *Dnmt3l* suggesting no canonical ∼50 kDa DNMT3L expression in MSCs. However, a potential novel isoform of *Dnmt3l* transcript starting from exon 9a can be observed to be expressed in low level in wild type but not *Dnmt3l* KO mice derived BM-MSCs. In contrast, 3 *Dnmt3l* isoforms that were described in adult testes previously all starting from exon 9b. **(B)** The canonical stem cell form of ∼50 kDa DNMT3L expression is highly expressed in ES cells detected by Western blotting. However, even under overexposure, there were no detectable DNMT3L signals at early passage (P2, P3, and P4) of MSCs. **(C)** Possibility of small DNMT3L (estimated to be 126 AA of around 20 kDa) expression has not been excluded but no distinct bands may be observed from our Western analysis of the MSC samples.

We observed very strikingly reduced CFU-F and osteogenesis deficit of MSCs isolated from *Dnmt3l* KO mice (Figure 1B). The intuitive explanation could be that DNMT3L is expressed and plays a direct role in cultured MSCs when the cells are out of their endogenous niches. However, the expression of DNMT3L in MSCs has not been documented. We performed strand-specific RNA sequencing analysis for undifferentiated WT and *Dnmt3l* KO MSCs at passage 4, as well as for the differentiating cells of the two genotypes 3 days after osteogenic induction. We confirmed that *Dnmt3l* was not expressed in *Dnmt3l* KO MSCs. However, low levels of *Dnmt3l* associated sequences could be identified from undifferentiated and differentiating wild type BM-MSCs (Figure 2A). Interestingly, all of the *Dnmt3l* RNA sequences from WT MSCs mapped to exon 9a onward, missing exons 1–8 (Figure 2A). The absence of exons 1–8 and the presence of exon 9a–10–11–12–13 of the *Dnmt3l* transcript in WT BM-MSCs were supported by our RT-qPCR analysis with various primer pairs (data not shown). Three alternative transcripts of *Dnmt3l* starting from exon 9b were described during sperm development (Shovlin et al., 2007). However, a potential isoform starting from exon 9a instead of exon 9b has not been previously described.

On the protein level, we could not detect the canonical ∼50 kDa DNMT3L protein (Figure 2B), consistent with the lack of RNA expression from exons 1–8. Neither could we detect distinct immunoblotting signals of the estimated 126-AA-long protein from the possible *Dnmt3l* isoform starting from exon 9a (Figure 2C). We have not excluded the possibility that this exon 9a initiated *Dnmt3l* RNA isoform may have a function. Our preliminary RT-qPCR data demonstrated higher *Dnmt3l* expression from the 3′-end exons in EMSCs than in BM-MSCs, associated with more severe potency defects in *Dnmt3l* EMSCs.

### Transcriptome Analysis of Undifferentiated and Differentiating MSCs Derived From *Dnmt3l* KO Mice Compared to Those of Wild-Type Littermates

**FIGURE 3:**
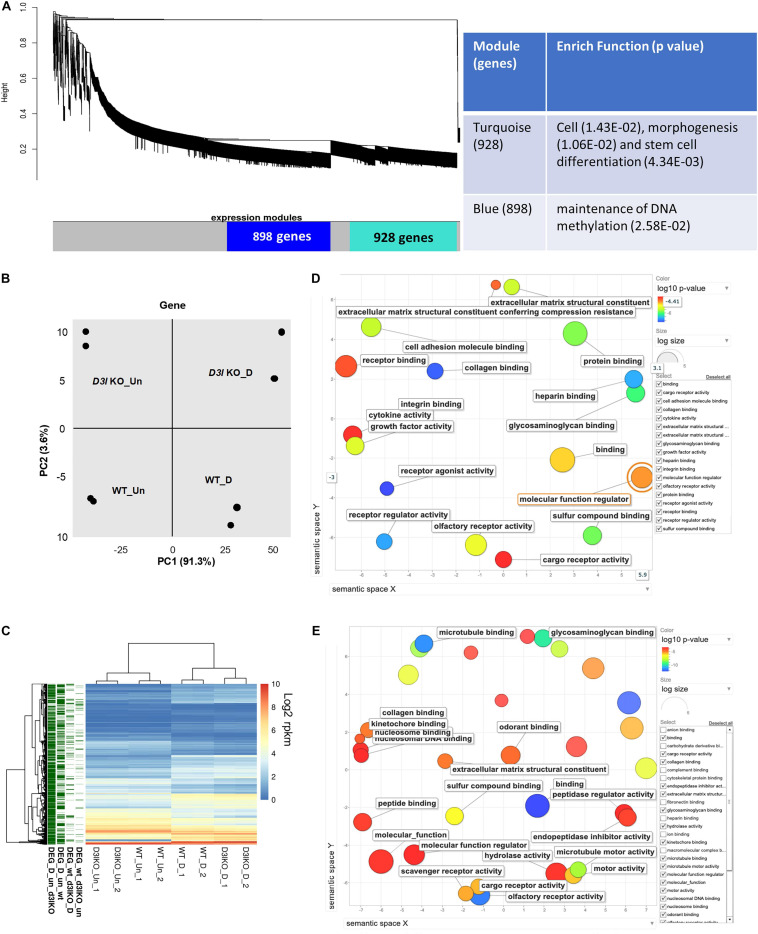
Analyses of *Dnmt3l* genotype-specific differentially expressed genes (DEGs) in undifferentiated and differentiating MSCs on day 3 of osteogenic induction. **(A)** Weighted correlation network analysis was performed, with the two major gene modules marked in turquoise and blue. The first (turquoise block) module contains 928 genes enriched in cell, morphogenesis and stem cell differentiation. The other module has 898 genes that are categorized as maintenance of DNA methylation. **(B)** Principal component analysis (PCA) of gene expression. **(C)** Each row represents a DEG. Four groups of DEGs are shown on the left and marked in green. Most DEGs highlight the transition during differentiation. There are also differences between the WT and *Dnmt3l* KO MSCs, indicating the contribution of DNMT3L to these differences. The heatmaps illustrate the expression patterns of genes that are modulated by either differentiation or the DNMT3L effect. DEG_wt_d3l_un: DEGs between WT and *Dnmt3l* KO in undifferentiated MSCs; DEG_wt_d3lKO_D: DEGs between WT and *Dnmt3l* KO in differentiating MSCs; DEG_D_un_wt: DEGs between undifferentiated and differentiating MSCs derived from WT mice; and DEG_D_un_d3lKO: DEGs between undifferentiated and differentiating MSCs in *Dnmt3l* KO mice-derived MSCs. **(D)** Gene ontology analysis of DEGs between WT and *Dnmt3l* KO undifferentiated MSCs. **(E)** Gene ontology analysis of osteogenic differentiating (day 3 of osteogenic induction) DEGs between WT and *Dnmt3l* KO MSCs. All GO terms presented on the bubble plots have an adjusted *p*-value of <0.05.

In order to gain insight into the mechanistic significance of the *in vitro* osteogenesis defects in *Dnmt3l* KO MSC, we performed WGCNA, an unbiased and unsupervised analysis that identifies modules corresponding to clusters of co-expressed transcripts among multiple transcriptomes. The results showed two major gene modules (Figure 3A, marked by turquoise and blue). The first module contains 928 genes enriched in “cell, morphogenesis and stem cell differentiation” by gene ontology analysis. The second module has 898 genes that are specifically associated with “the maintenance of DNA methylation.” In addition, PCA revealed four distinct groups signifying that cell differentiation and *Dnmt3l* genotype contribute to significant transcriptome changes (Figure 3B).

In addition to global gene expression patterns observed by PCA, DEG (at least log_2_ fold change > 1 with FDR < 0.05) between undifferentiated and differentiating MSCs, or between different *Dnmt3l* genotypes of the same differentiation stage, were indicated in the dendrogram (Figure 3C). In Figure 3C, each row presents a DEG. Approximately 300 genes (Figure 3C, DEG_wt_*d3l*KO_un group) were differentially expressed in *Dnmt3l* KO MSCs in the undifferentiated state, of which 106 were upregulated and 202 were downregulated. These DEGs were highly correlated to receptor binding, cytokine activity, cargo receptor activity, and ECM structural constituent, which may directly or indirectly influence the MSC differentiation potential upon induction of differentiation (Figure 3D). In addition, some of these DEGs were overlapped with signature genes associated with bone related phenotypes as summarized in the MGI mammalian phenotypes database (Table 1). For example, *Postn* encodes the matricellular protein POSTN, which is downregulated in *Dnmt3l* KO MSCs. *Postn* is transcriptionally activated by RUNX2 in the MC3T3-E1 osteoblast precursor cell line, suggesting that POSTN, also known as OSF-2, is involved in early osteoblast development (Stock et al., 2004). This gene is highlighted in the categories of abnormal long bone morphology (MP:0003723), short femur (MP:0003109), and short tibia (MP:0002764). Previous studies also demonstrated that POSTN may play a crucial role in bone formation and homeostasis (Bonnet et al., 2012; Merle and Garnero, 2012; Zhang et al., 2017), which is highly correlated to the jeopardized osteogenic differentiation ability observed in *Dnmt3l* KO MSCs.

**TABLE 1:** MGI mammalian phenotypic identification of undifferentiated DEGs between wild-type and *Dnmt3l* KO MSCs by enrichment analysis.

	*P*-value	Adjusted *p*-value
Decreased body weight (MP:0001262)	0.001719	0.128
Abnormal macrophage physiology (MP:0002451)	0.0009388	0.1133
Abnormal long bone morphology (MP:0003723)	0.0003274	0.1133
Short femur (MP:0003109)	0.0004068	0.1133
Short radius (MP:0004355)	0.0002514	0.1133
Abnormal ureter morphology (MP:0000534)	0.0002751	0.1133
Short tibia (MP:0002764)	0.001353	0.1205
Abnormal neuromuscular synapse morphology (MP:0001053)	0.0005063	0.1133
Decreased placenta weight (MP:0004921)	0.0004709	0.1133
Abnormal forelimb morphology (MP:0000550)	0.0008594	0.1133

Upon comparing transcriptomes of MSCs derived from *Dnmt3l* KO and WT mice after 3 days of induction toward osteogenesis, a more significant change was observed (*n* = 567 DEGs, 239 genes were upregulated and 328 genes were downregulated) (Figure 3C, DEG_wt_*d3l*KO_D group). We noticed a clear enrichment of these DEGs in molecular binding related properties, including nucleosome and nucleosomal DNA, peptide, kinetochore, odorant, and collagen binding, as well as genes associated with general molecular function regulation. In addition, with enrichment of DEGs in GO terms associated with collagen binding, hydrolase activity and ECM structural constituent, we suggest that MSCs derived from *Dnmt3l* KO mice may have suboptimal modulatory activities and responsiveness toward ECMs (Figure 3E).

With candidate approach, we also have observed the expression profile of several other bone related biomarkers, including *Sp7*, *Col1a1* and *Col1a2*, from our sequencing datasets. *Dnmt3l* genotype associated, statistically significant differences were observed in all of these genes at day 3 of *in vitro* MSC differentiation toward osteoblast, while significant differences can be found in *Col1a1 and Col1a2*, as well as the aforementioned *Postn* genes in MSCs of different *Dnmt3l* genotype even before differentiation (Supplementary Figure 4).

### Searching for Potential DNMT3L-Mediated Epigenetic Memory From ES Cells

Given DNMT3L protein was not detected in MSCs, we explored the possibility of DNMT3L-mediated epigenetic modulation in pluripotent stem cells; we hypothesized that the DNMTs may exhibit a long term effect that could alter the differentiation potential of the cultured MSCs *in vitro*. We re-analyzed publicly available DNMT3L ChIP-Seq (GSE49178), DNMT3L KD ES cells gene expression array (GSE44643) and MeDIP sequencing (GSE44642) data (Neri et al., 2013).

**FIGURE 4:**
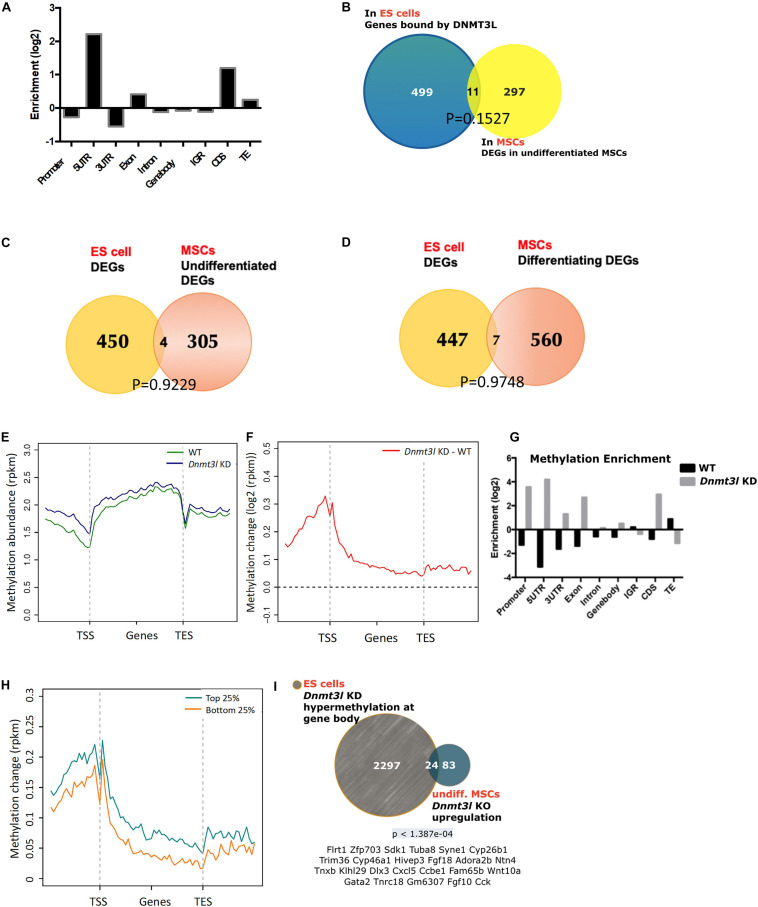
Possibility of DNMT3L-mediated legacy and the DNA methylation level contributing to the compromised self-renewal and differentiation potential of *Dnmt3l* KO MSCs. **(A)** Enriched DNMT3L binding in ES cells is observed in the 5′UTR and coding region of coding genes. **(B)** Only 11 out of 308 DEGs between undifferentiated MSCs derived from WT and *Dnmt3l* KO mice are direct DNMT3L binding targets in ES cells. **(C)** Only four genes are differentially expressed in both ES cells and undifferentiated MSCs between the WT and *Dnmt3l* mutant cells (*Dnmt3l* KO MSCs and *Dnmt3l* KD ESCs); **(D)** Only seven genes are differentially expressed in both ES cells and differentiating MSCs between WT and *Dnmt3l* mutants. **(E)** DNA methylation level in WT and *Dnmt3l* KD ES cells are profiled for all genes as well as their immediate upstream and downstream regions. **(F)** The methylation regions in *Dnmt3l* KD ES cells are enriched in the 5′UTR and coding sequence. **(G)** Differential gene methylation between WT and *Dnmt3l* KD ES cells. Greater enrichment on TEs in WT ES cells are observed, indicating that DNMT3L facilitates *de novo* methylation in these sequences. **(H)** The difference in gene methylation between WT and *Dnmt3l* KD in high/low expression genes. **(I)** Twenty-four out of 107 genes upregulated in Dnmt3l KO MSCs are overlapped with hypermethylated genes in ES cells with *Dnmt3l* knock down. The *p*-value calculated by hypergeometric test is very low, indicating that these 24 up-regulated genes in *Dnmt3l* KO MSCs are not due to random overlapping in highly methylated ES cells.

To understand which genes are directly regulated by DNMT3L, we categorized DNMT3L binding sites in gene bodies, promoters or repeat sequences from DNMT3L ChIP-Seq data from ES cells. We observed that DNMT3L binding sites were enriched in 5′-UTRs, coding regions and TEs (Figure 4A). To verify the lasting impact of DNMT3L from ES cells to MSCs, we first sought to identify the overlap between DNMT3L binding sites in ES cells and DEGs between the WT and *Dnmt3l* KO MSCs. However, in both undifferentiated and differentiating MSCs, only 11 *Dnmt3l* genotype associated DEGs were also associated with DNMT3L binding in ES cells (Figure 4B and Supplementary Figure 5); suggesting most DEGs in MSCs were not the direct targets of DNMT3L in ESCs.

To investigate whether DNMT3L-dependent gene expression patterns carry over from ES cells to MSCs, DEGs from an expression array of ES cells were compared to MSC RNA-Sequencing data. Only 4 out of 309 genes were differentially expressed in the *Dnmt3l* KD ES cells and undifferentiated *Dnmt3l* KO MSCs (Figure 4C), and 7 out of 567 DEGs were identified in differentiating MSCs (Figure 4D), indicating that the role of DNMT3L legacy from ES cells on MSC properties is not a primary effect in gene expression.

DNMT3L is a well-known epigenetic cofactor, functioning within the *de novo* DNA methylation machinery. With the faithful memory of DNA methylation after cell division, alterations in *Dnmt3l* genotype-dependent DNA methylation level in ES cells could therefore be one of the most promising long-lasting epigenomic marks that are potentially responsible for the compromised *Dnmt3l* KO MSCs properties. To understand the influence of DNMT3L on DNA methylation, we analyzed the DNA methylation patterns of WT and *Dnmt3l* KD ES cells. According to the metagene plots, the methylation of *Dnmt3l* KD ES cells was generally higher than WT cells around genes, particularly at the promoter region (Figures 4E,F), and the same result was observed in the enrichment analysis (Figure 4G). We further found that these changes in DNA methylation occurred in genes from ES are associated with increased expression (between *Dnmt3l* KO and WT) in MSC (Figure 4H), indicating a positive correlation between changes in DNMT3L-induced methylation in ES cells and gene expression in MSCs, as a potential signal of DNMT3L legacy.

We further clarified whether the differential gene expressions in *in vitro* cultured *Dnmt3l* KO and WT MSCs, a cell type no longer expressing DNMT3L, may have association with DNMT3L-dependent DNA methylation mark in ES cells. Apart from the epigenomic modulation of regulatory sequences, DNA methylation on actively transcribed genes, had also been demonstrated (Bender et al., 1999).

We observed that 24 genes were upregulated in *Dnmt3l* KO MSCs and hypermethylated at the gene bodies in Dnmt3l KD ES cells (Figure 4I and more comprehensive analysis summarized in Supplementary Figure 6), with a very low *p*-value < 1.387e-04, indicating the significant overlap based on the hypergeometric distribution test. Of the 24 genes, some gene products have been reported to tie with skeletal development or homeostasis, with a few of them acting directly in MSCs to regulate osteogenic differentiation, namely, HIEVP3 and GATA2 inhibiting BMP-induced osteogenesis (Tolkachov et al., 2018; Li et al., 2020), DLX3 being a negative modulator (Levi and Gitton, 2014), while FGF18 and Wnt10a providing stimulatory signal (Charoenlarp et al., 2017; Jing et al., 2018). Those results suggest that the occurrence of DNMT3L-mediated epigenetic legacy, if any, may be observed in limited genes in cultured MSCs.

### Altered Transposable Element Expression Profile Suggesting Changes of Global Epigenomic Landscape

**FIGURE 5:**
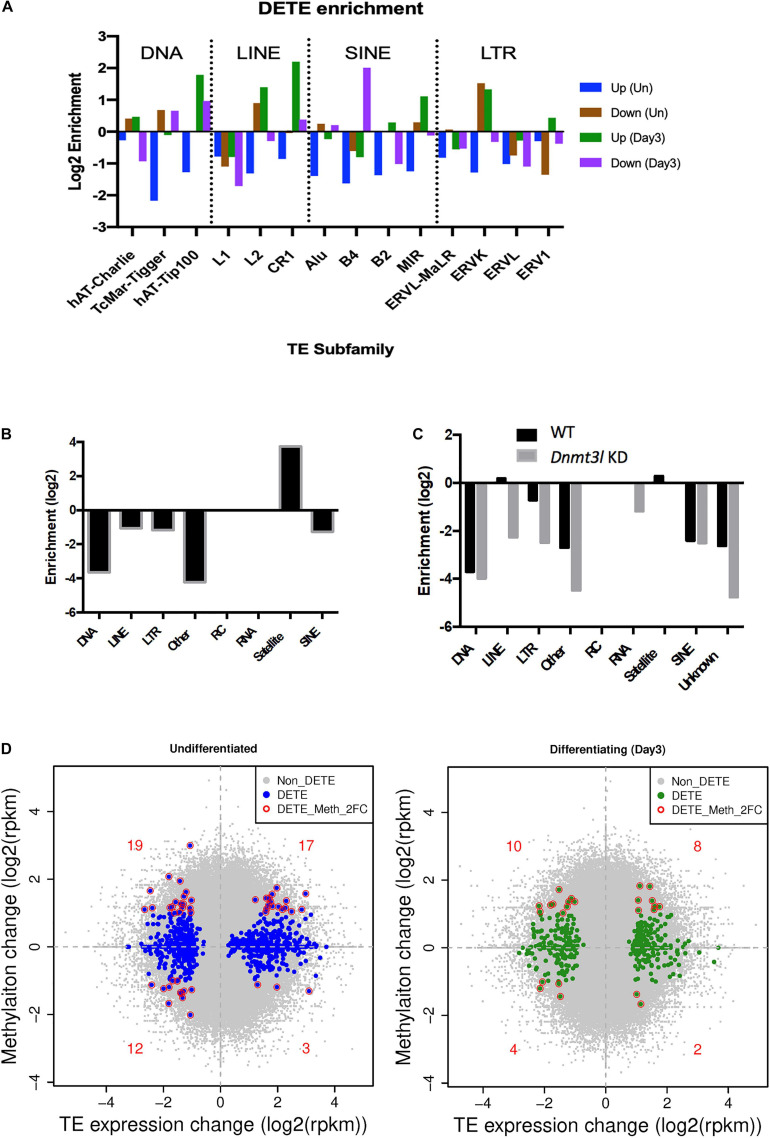
*Dnmt3l* genotype associated differential TE expressions and DNA methylation levels in MSCs. **(A)** Significant changes of TE expression patterns were observed in *Dnmt3l^– /–^* undifferentiated and differentiating MSCs, compared to wild type MSCs. Blue, upregulated TEs in undifferentiated *Dnmt3l* KO MSCs, UP (Un). Brown, downregulated TEs in undifferentiated *Dnmt3l* KO MSCs, Down (Un). Green and purple indicate upregulated and downregulated TEs in differentiating MSCs at day 3 of osteogenesis induction, UP (Day3) and Down (Day3), respectively. **(B)** The DNA methylation is obvious low at TE regions in ES cells. In addition to repeat sequences, DNMT3L binding regions are mostly enriched in satellites in ES cells. **(C)** DNMT3L associated differentially methylated TEs in ES cells **(D)** Blue dots indicate *Dnmt3l* KO genotype associated differentially expressed TEs in undifferentiated MSCs (left); Green dots indicate *Dnmt3l* KO genotype associated differentially expressed TEs in differentiating MSCs (right); red circled dots indicate differentially expressed TE with a ≥2-fold change in methylation in *Dnmt3l* KD ES cells.

Because of the known transposon element (TE) silencing activity of DNMT3L (Bourc’his and Bestor, 2004), we assessed the possibility of correlating specific families of deregulated TEs to the impaired properties of *Dnmt3l* KO MSCs. The DETEs with four-fold changes were subjected to unsupervised heat map analysis (Supplementary Figure 7A). Genotype-dependent differences (four-fold changes) were manifested in an undifferentiated but not at differentiating state based on PCA analysis (Supplementary Figure 7B). Even without considering vast copy numbers of each TE sequences, there are more DETEs between WT and *Dnmt3l* KO genotypes in undifferentiated MSCs than the number of DEGs (Supplementary Figure 7C). DETEs populated in specific subfamilies such as DNA transposon, LINE, SINE and LTR, suggesting the accumulative effects from DNMT3L targeting these four subfamilies of TEs (Figure 5A). The significant reduction of upregulated TEs from undifferentiated *Dnmt3l* KO MSCs across many TE families (blue bars from Figure 5A) indicated possible epigenomic signature changes across many genomic loci from this cell type. In the differentiating MSCs, DETEs were enriched in the subfamilies of DNA transposon (hAT-Tip 100), LINEs (L2 and CR1), SINE (B4) and ERVK of the LTR family (green and purple bars in Figure 5A).

With the known property of DNMT3L in TE modulation, we also analyzed the profile of DNMT3L associated DNA methylation marks from ES cells in relation to TE expressions in cultured MSCs of different *Dnmt3l* genotype. The DNA methylation is relatively low at most TE regions in ES cells. *Dnmt3l* KD induced further reduction was particularly enriched in LINE and LTR retrotransposons in ES cells (Figures 5B,C). In addition, we investigated the potential role of *Dnmt3l* in undifferentiated and differentiating MSCs using differentially expressed analyses of TEs. The direct correlation between changes in DNA methylation and gene expression of TEs (Figure 5D) did not provide clear evidence of an association in a specific direction. However, our results displayed that the change of global epigenomic landscapes are associated with the altered expression of TEs. These affected TEs or their neighboring genes in *Dnmt3l* KO MSCs may contribute to the observed osteogenesis defect phenotype *in vitro*.

### Bone Morphology Analysis *in vivo*

In contrast to the strong osteogenesis defects *in vitro*, we did not observe obvious differences in trabecular bone phenotype in 5-months-old *Dnmt3l* KO females (Supplementary Figure 8). In 23-months-old wild type female mice, we can indeed observe the aging induced reductions in trabecular number, connectivity density, percent bone volume, which give higher risk for vertebral fracture, as well as increase in SMI and reductions in bone thickness and bone surface density expected for aging mice (Qiu et al., 2006; Ostertag et al., 2009; Chen et al., 2013). However, we did not observe the expected osteoporosis related symptoms in *Dnmt3l* KO aging female mice. In contrast, a protective effect was observed in the parameters including connectivity density and trabecular thickness (Supplementary Figure 8).

In an attempt to induce accelerated aging, we introduced high fat diet and time-zone shifting model in 6-month-old *Dnmt3l* KO male mice and their littermates. The light-dark cycle was shifted 6 h forward each week, for six times in total. Circadian disruption has been associated with metabolism defect and aging-like phenotype (Sato et al., 2017). With the combined jet-lag paradigm and high fat diet treatment, we no longer observed *Dnmt3l* genotype specific differences in bone properties (Supplementary Figure 9). We concluded that *Dnmt3l* KO male mice have stronger response to lifestyle intervention-induced trabecular bone mass reduction.

## Discussion

Mesenchymal stem/stromal cells hold promises for regenerative medical applications either by cell transplantation directly or by applying their secretory products (Madrigal et al., 2014; Han et al., 2019). *In vitro* amplification is needed for most of these practices. Various advancements have made to maintain and amplify MSCs in culture condition (Sotiropoulou et al., 2006). However, genotype effect cannot be easily fixed. Apart from infertility, *Dnmt3l* KO mice does not cause any other obvious defects. It is therefore a surprise for us to see obvious osteogenesis impairment of MSCs derived from *Dnmt3l* KO in this current study, especially when DNMT3L protein expression cannot be detected in these cell type. Although we cannot exclude the possibility that a small population of MSCs may express *Dnmt3l*, it is notable that the impaired CFU-F forming and osteogenic activities occurred in global population of MSCs and not the small population of *Dnmt3l*-expressing MSCs. The current study also identified a potential novel isoform of *Dnmt3l* transcript starting from exon 9a. Our preliminary RT-qPCR data suggested higher expression from the 3′-end of *Dnmt3l* in EMSCs than in BM-MSCs, associated with more severe potency defects in *Dnmt3l* EMSCs. The possibility for this partial transcript of *Dnmt3l* exon 9a onward being functional could not be excluded at this stage.

**FIGURE 6:**
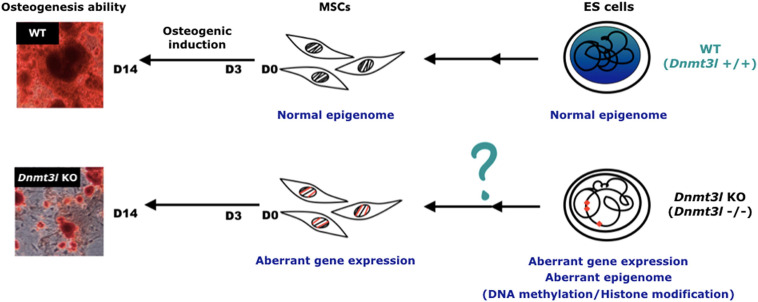
Hypothesis of DNMT3L-mediated epigenome legacy from embryonic stem cells influencing MSC properties. We propose that the expression of DNMT3L in ES cells is essential to maintain the normal epigenome. Some of these DNMT3L associated epigenomic landscape may be carried over all the way from pluripotent stem cells to influence isolated MSC properties from adult mice, and their self-renewal and differentiation abilities *in vitro*.

However, it is still more plausible that the differences in the cultured MSCs was due to an accumulative cellular memory pre-deposited in DNMT3L expressing progenitor cells. DNMT3L is predominantly expressed in ES cells. We have recently demonstrated that transient ectopic DNMT3L expression in fibroblast is sufficient to introduce long-term effect on epigenome and cell proliferation property, over 40 passages after the ectopic DNMT3L is silenced (Yu et al., 2020), speculating that transiently expressed DNMT3L in ES cells or other progenitor cells, may also have long lasting effects that protect MSCs from premature senescence. The hypothesis of “DNMT3L-mediated epigenetic legacy” (Figure 6) assumes that DNMT3L sustains a normal epigenome or gene expression profile in ES cells. Without DNMT3L expression in the pluripotent stem cells of *Dnmt3l* KO preimplantation embryos, some of the aberrant epigenetic signatures may be carried over to somatic progenitor cells, and therefore compromising the property of *Dnmt3l* KO MSCs under *in vitro* culture and differentiation condition. When comparing DNMT3L binding sites and DNMT3L associated gene expression pattern and methylation status in ES cells, with the DEGs between MSCs from *Dnmt3l* KO and wild type littermates. There is no strong correlation between DNMT3L bound regions or DNMT3L associated DEGs in ES cells, with *Dnmt3l* genotype related DEGs in MSCs. Interestingly, DNA methylation at the gene body and promoter regions is most affected in *Dnmt3l* KD ES cells. Our results showed that gene bodies contain the most overlapped targets, with the effect of DNA methylation potentially carried over from ES cells into MSCs (Figure 4I). Among the 107 DEGs between undifferentiated *Dnmt3l* KO and wild type MSCs, 24 bears DNMT3L associated DNA methylation on their gene bodies in ES cells. Several of these 24 genes have direct or indirect implications in bone morphogenesis or homeostasis. For example, HIVEP3 (also called Schnurri-3, Shn3) inhibits BMP9-induced osteogenic differentiation from human amniotic MSCs (Tolkachov et al., 2018; Li et al., 2020), and GATA2 is implicated in interfering BMP2-induced SMAD signaling and thus inhibiting osteoblast differentiation demonstrated by an ectopic expression experiment (Tolkachov et al., 2018; Li et al., 2020). FGF18 stimulates rat and mouse MSCs for their osteogenic differentiation (Charoenlarp et al., 2017; Jing et al., 2018). DLX3 serves as a negative regulator of osteogenesis and contributes to bone homeostasis with other transcription factors, such as DLX5 (Levi and Gitton, 2014). Also, Cyp26b1 has effect in osteoblast and Cxcl5 functions for mobilizing HSC away from BM. Although these last two factors may not directly contribute to the phenotype observed in our MSC *in vitro* osteogenesis impairment phenotype, they provide implications for the miscommunication between the niche MSCs and the DNMT3L expressing HSCs, the progenitor of osteoclast cells. Although there are only limited numbers of genes having correlation between their DNMT3L associated DNA methylation in ESCs and their *Dnmt3l* genotype associated expression patterns in MSCs, the several highlighted genes may be important enough to contribute in part to the osteogenesis defects observed. In addition, potential DNMT3L associated legacy from progenitor cells or niche interacting defects may go beyond DNA methylation level, and beyond coding genes. The fact that more TEs related sequences are deregulated in cultured *Dnmt3l* KO MSCs, associated to the deregulation of methylation landscape in *Dnmt3l* KD ESCs, indirectly support this notion.

The serious loss of osteogenesis ability and retained adipogenesis ability of the cultured *Dnmt3l* KO MSCs out of their endogenous niche resembled the tilted osteogenesis to adipogenesis balance of aging MSCs (Yang et al., 2018). Several mechanisms may simultaneously contribute to MSC aging and senescence related phenomena, including telomere shortening, mitochondrial dysfunction resulting from oxidative stress, the accumulation of DNA damage, and genome wide shifting of the epigenetic modification landscape (Li et al., 2017). Whereas the physiological oxygen level in healthy human bone marrow is approximately 7.5% (Harrison et al., 2002; Jagannathan et al., 2016), conventional cell culture systems use roughly 19% oxygen, which may pose oxidative stress to MSCs, leading to mitochondrial dysfunction and cellular senescence. Indeed, recent studies indicated that hypoxic conditions (1% oxygen) prevent MSCs from entering senescence via the HIF-1α pathway (Tsai et al., 2011). However, osteogenesis may be inhibited under this extreme hypoxic condition (Yang et al., 2011). Interestingly, MSCs consume more oxygen for increased mitochondrial oxidative phosphorylation and exhibit downregulated levels of HIF-1α during osteogenesis, whereas glycolysis activity remains consistent (Shum et al., 2016). It has been shown that HIF-1α requires pyruvate for cancer cells under aerobic conditions (Lu et al., 2002). Accordingly, we observed that the levels of glyceraldehyde 3-phpsphate dehydrogenase (*GAPDH)*, a key player in pyruvate metabolism of glycolysis, drastically fluctuate between undifferentiated and differentiating MSCs (data not shown) despite culturing MSCs under 19% oxygen and with pyruvate. Although the detailed mechanisms for the failure of osteogenic induction in *Dnmt3l* KO MSCs require much further study, it is possible that metabolic defect could be one of the legacies pre-deposited from DNMT3L-expressing progenitors. Our ongoing study concerning dietary alteration-associated effects in paired *Dnmt3l* KO and WT littermates also shows similar implications.

Our previous study demonstrated that MEFs derived from *Dnmt3l* KO embryos enter senescence prematurely. This phenomenon is associated with a reduction of suppressive H3K9me3 and H3K27me3 marks and the premature extrusion of HDAC1 to the cytoplasm (Liao et al., 2015). On the other hand, ectopic expression of DNMT3L in aging MEFs significantly increases the nuclear localization of HDAC1. In addition, exogenous DNMT3L is sufficient to recruit DNMT3A/KAP1/SETDB1/HDAC1 to both newly infected retroviruses and some endogenous retroviruses by inducing H3K9me3 in aging MEFs (Kao et al., 2014). As non-canonical bivalent H3K4me3/H3K9me3 signature was observed on adipogenic determining genes, *Cebp-alpha* and *Ppar-gamma* (Matsumura et al., 2015) in MSC cell line, we looked into whether the SETDB1-dependent repressive H3K9me3 marks on those adipogenic master regulator genes accounted for a possible DNMT3L associated epigenetic legacy in MSCs that prevented premature determination of adipogenic fate. Although we have not obtained any conclusion from H3K9me3 ChIP-qPCR experiments, lacks of *Cebp-alpha* and *Ppar-gamma* upregulations in undifferentiated *Dnmt3l* KO MSCs or their osteogenic differentiating derivatives suggest the impaired non-canonical bivalent mark on adiogenic genes is unlikely the primary reason behind the titled osteogenesis to adipogenesis balance observed in *Dnmt3l* KO MSCs *in vitro*.

Despite the serious osteogenesis impairment of *Dnmt3l* KO MSCs *in vitro*, counterintuitively, does not correlate to strong bone formation defects or osteoporosis phenotype *in vivo*. One may consider the balance between activities of osteoblasts and osteoclasts *in vivo* for the bone density phenotype. Since DNMT3L has been shown to be expressed in hematopoietic stem cells (HSCs), the progenitor cells of osteoclasts (Fierro et al., 2017), DNMT3L deficiency in HSCs may potentially contribute to reduced osteoclast function and hence the increased bone density observed in *Dnmt3l* KO mice. MSCs are also determined as one of the cell types building HSC niche (Ehninger and Trumpp, 2011), suggesting potential intercommunication between these two cell lineages. Interestingly, expression of the *Cxcl5* gene that encodes a chemokine to induce mobilization of HSC from BM (Yoon et al., 2012) was elevated in *Dnmt3l* KO MSCs, whereas expression of the factors for HSC maintenance, *Angpt1* and *Cxcl12* were significantly decreased (Supplementary Table 3). Altogether, in *Dnmt3l* KO mice, the defective osteogenesis of MSCs as disclosed in our *in vitro* study, combined with the proposed inefficiency of bone breakdown, would give rise to only subtle, if any, phenotypes *in vivo* concerning the bone homeostasis. The in-depth study of DNMT3L function in HSCs and their derivatives would be another important episode for understanding the function and properties of this important epigenomic modulator DNMT3L beyond its highly expressing pluripotent stem cells and germ cells.

We demonstrated in this study that long term effects on cell fate and property can be induced by transient expression or deficiency of epigenetic modifiers and cofactors. These effects may even be amplified from spreading of the epigenetic alternations through excessive cell proliferations *in vitro* or *in vivo*. This is particularly important for the cell-based regenerative medicine when transient exposure of stem cells under aberrant condition would have long lasting effect that may not be perceived before transplantation, but conveying long-term risk thereafter.

## Materials and Methods

### Animals

Due to the infertility phenotype for both male and female *Dnmt3l* KO mice, the Dnmt3l^+/tmEnls^ mutant allele was maintained as heterozygous colony. *Dnmt3l* KO mice and *Dnmt3l*^+/+^ littermate controls were generated from intercrossing mice with the heterozygous Dnmt3l^+/tmEnls^ mutant allele (Hata et al., 2002) in a C57BL/6 genetic background. In average, one *Dnmt3l^–/–^* KO mice and one *Dnmt3l*^+/+^ littermate control from one gender were generated from one litter. Female mice (8–16 weeks old) were euthanized to harvest MSCs for experiments. The care of mice and the experimental procedures involving animals were approved by the Institutional Animal Care and Use Committee (IACUC) of National Taiwan University (approval number NTU-104-EL-00031).

### Isolation of Mouse Bone Marrow MSCs

For each experiments, we collected bone marrow MSCs from three *Dnmt3l* KO mice of the same gender and age, as well as the counterparts from the three *Dnmt3l*^+/+^ littermate controls, to prepare mixed MSCs from each genotype. The procedure used to isolate mouse bone marrow MSCs was previously described (Cheng et al., 2012, 2014; Lee et al., 2017). Briefly, the femur and tibia were separated from the mouse after euthanasia by quickly removing the muscle and connective tissue with forceps and scissors. A 23-gauge needle containing 12 mL of culture medium [αMEM (Gibco, Grand Island, NY, United States) supplemented with 20% FBS (HyClone, Logan, UT, United States) and antibiotics (100 U/mL penicillin and 100 μg/mL streptomycin; Gibco)] was used to flush out femur bone marrow. A similar procedure was performed with a 26-gauge needle and 6 mL of culture medium to acquire tibia bone marrow. The bone marrow and minced femur and tibia were cultured in 100-mm Petri dishes (TPP, Trasadingen, CH, Switzerland). After 9 days of culturing with medium changes every 3 days to deplete non-adherent cells, adherent cells were detached with 0.25% trypsin/EDTA (Gibco) for 5 min in an incubator. Cells were then transferred to a 70-μm nylon cell strainer (Falcon, Corning, NY, United States) to remove cell clumps. MSCs were isolated using a MACS system (Miltenyi Biotec, Teterow, DE, Germany) with CD11b (Miltenyi Biotec) and CD45 (Miltenyi Biotec) microbead antibody double-negative selection according to the manufacturer’s protocol to avoid hematopoietic lineage contamination. Briefly, 10^7^ cells were resuspended in 10 μL of CD11b antibody, 10 μL of CD45 antibody and 180 μL of MACS^®^ running buffer (Miltenyi Biotec), and the cells were left to react for 15 min at 4°C. One microliter of running buffer was added to stop the reaction, and the cells were centrifuged for 5 min at 1200 rpm. Subsequently, the supernatant was removed, and another 1 mL of running buffer was added to resuspend the cells. A MACS^®^ LD column (Miltenyi Biotec) was inserted into a MidiMACS^TM^ separator (Miltenyi Biotec), and a collection tube was placed under the column to harvest MSCs. The column was rinsed with 2 mL of running buffer. Afterward, the cells were transferred into the column. When the column was about to dry out, 1 mL of running buffer was added to wash out unlabeled cells (i.e., non-hematopoietic cells). The procedure was repeated, and enriched MSCs were harvested from the effluent. After 5 min of centrifugation at 1200 rpm, running buffer was removed and replaced with culture medium. MSCs were seeded at a density of 5 × 10^4^ cells per cm^2^.

### Isolation of an MSC Subtype: Epiphysis-Derived Mesenchymal Stem/Stromal Cells (EMSCs)

The procedure used to harvest the EMSC subtype was based on our previous report (Cheng et al., 2012). We isolated the femurs and tibias of mice and thoroughly removed the adherent soft tissues. The epiphyses were then removed using shears and rinsing out of any possible bone marrow fluids with culture medium. The cleaned intact epiphyses (without mincing) were then transferred to 22.1 cm^2^ culture dishes (TPP) and cultured in complete medium composed of MEM alpha (Sigma-Aldrich) supplemented with 20% FBS (HyClone), 2 mM L-glutamine (100 U/ml penicillin, and 100 μg/ml streptomycin; Invitrogen). The cells were incubated under a humidified atmosphere containing 95% air and 5% CO_2_ at 37°C. The non-adherent cells were removed by changing the medium every 3 days. After 11 days, the primary culture reached approximately 70% confluence, and the cells were detached with 0.25% trypsin/0.1 mM EDTA (trypsin/EDTA; Invitrogen) for 1 min at 37°C. Subsequently, the reaction was stopped by adding complete medium. The cells that could not be lifted within 1 min were discarded. The detached cells were centrifuged at 400 × *g* for 5 min, resuspended in the complete medium, and subjected to negative selection using the CD11b and CD45 antibodies as described in the BM-MSC isolation section above.

### Colony-Forming Unit Assay

One or two thousand cells from passage 3 were seeded into 6-well plates (TPP). After 9 days of culture, cells were fixed with methanol (J. T. Baker, Center Valley, PA, United States) and visualized by Giemsa stain (Gibco). Colonies greater than 2 mm in diameter were defined as valid.

### Cell Growth Assay

The PDT was determined to assess the growth of MSCs. In brief, *Dnmt3l* KO MSCs and wild type MSCs from passage 3 were seeded for 33 wells with a density of 2 × 10^4^ cells per well in 12-well plate. These cells were allowed to adhere for 24 h at 37°C under an atmosphere with 95% air and 5% CO_2_. Cells from three wells of each genotype were isolated and evaluated by trypan blue staining each day for 11 days to calculate the number of living cells.

### Osteogenic Differentiation

Osteogenic induction medium was composed of 10% FBS-αMEM supplemented with 0.1 μM dexamethasone (Sigma-Aldrich, St. Louis, MO, United States), 10 mM β-glycerophosphate (Sigma-Aldrich) and 50 μM L-ascorbic acid 2-phosphate (Sigma-Aldrich) (Jaiswal et al., 1997). For each sample, 5 × 10^4^ cells per cm^2^ were seeded into a 6-well plate. After reaching confluence in 2 days, the cells were incubated with osteogenic induction medium. The medium was changed every 3 days, and analyses were performed after 14 days of induction. To assess calcium precipitation, the induction medium was removed, and the cells were washed once with PBS. Next, the cells were fixed with 10% formaldehyde (Sigma-Aldrich) for 10 min with gentle shaking, and after another wash with PBS, the cells were stained with 500 μL of 2% ARS (pH 4.1–4.3) (Sigma-Aldrich) for 15 min. After extensive PBS irrigation, the precipitate was dissolved in 500 μL of 10% cetylpyridinium chloride (Sigma-Aldrich) in 8 mM Na_2_PO_4_ (Sigma-Aldrich) and 1.5 mM KH_2_PO_4_ (Sigma-Aldrich). The absorbance at 550 nm was recorded, and quantification of calcium was determined using an optimal ARS standard curve. The optimal dilution was performed to ensure that the sample absorbance fell within the standard curve.

### RNA Extraction, Library Preparation and Sequencing

Total RNA was extracted using TRIzol reagent (Invitrogen, Carlsbad, CA, United States) and a RNeasy mini kit (Qiagen, Hilden, DE, Germany) following the manufacturer’s instructions. RNA was treated with DNase 1 (Qiagen) to prevent DNA contamination. The quality of total RNA was checked using an Agilent Bioanalyzer 2100 with an Agilent RNA 6000 Nano Kit, and the RIN of the total RNA samples were all greater than 8. We used 2.5 μg of total RNA as input for strand-specific RNA library preparation following the removal of rRNA using a Ribosomal Illumina Ribo-Zero Gold rRNA Removal Kit (Human/Mouse/Rat) (Illumina, San Diego, CA, United States). The rRNA-depleted RNA libraries were constructed using an Illumina TruSeq Stranded mRNA Sample Prep kit following the user guide at the fragment RNA step. The libraries were sequenced using an Illumina HiSeq 2500 system with Rapid run mode (50-bp single-end reads). Each sample was sequenced to obtain approximately 70.7 M raw reads.

### RNA-Seq Analysis

The mRNA reads were strand-specifically aligned to the mouse reference genome (mm10) using Tophat2 (Kim et al., 2013; Trapnell et al., 2013), with the gene annotation downloaded from iGenome. The reads per gene counts were generated using Cuffdiff (Trapnell et al., 2013), which was also used to identify DEGs between samples using a ≥2-fold change in expression and a FDR less than 5%. The transposon annotation was downloaded from UCSC (Karolchik et al., 2004). The expression counts of transposons were generated using multiBamCov from BEDtools (Quinlan and Hall, 2010), and a transposon was considered to be expressed if it was expressed in at least one or more samples. The normalization of sequencing depth across samples and differential transposon expression analysis was performed using edgeR (Robinson et al., 2010) with a ≥2-fold change in expression and FDR 5%. The minimum expressed TE replicate had to have a minimum of 10 reads among all samples after normalizing for sequencing depth across samples.

### Weighted Gene Co-expression Network Analysis (WGCNA)

Weighted gene co-expression network analysis was performed using the R package for transcriptome data following the standard method described on the authors’ website (Langfelder and Horvath, 2008). This unsupervised and unbiased analysis identifies distinct co-expression gene modules by clustering transcripts with similar expression patterns across samples. Transcriptionally variable genes with an RPKM of ≥0.5 were selected for the analysis. These genes were hierarchically clustered based on a dissimilarity measure of topological overlap matrix. The resulting dendrogram was used for module detection (minimum size 100; height cutoff 0.23). Gene modules were labeled in unique colors, with unassigned genes labeled in gray.

### Bioinformatic Analysis on ChIP-Seq and MeDIP-Seq Datasets

The ChIP-Seq of DNMT3L from mouse ES cells was from GSE49178 (Neri et al., 2013), and the DNA methylation data (MeDIP-Seq) from mouse *Dnmt3l* KD ES cells was from GSE44642 (Neri et al., 2013). All reads were aligned to the mouse reference genome (mm10) using Bowtie2 (Langmead and Salzberg, 2012), with peaks called using MACS2 (Feng et al., 2012).

### Western Blot

Cells were homogenized and lysed in RIPA lysis buffer (EMD Millipore, Burlington, MA, United States) supplemented with 1 mM phenylmethylsulfonyl fluoride (Sigma-Aldrich) and protease inhibitor cocktail (Sigma-Aldrich). Lysate (5–10 μg per lane) were separated by sodium dodecyl sulfate–polyacrylamide gel electrophoresis (SDS–PAGE) and transferred to PVDF membranes (EMD Millipore). The membranes were blocked with 5% BSA (Sigma-Aldrich) in 1x PBST for 1 h at room temperature or 4°C overnight. The incubation with primary antibody anti-DNMT3L (E1Y7Q, Cell signaling technology, 1:1000 dilution), and anti-GAPDH (ab181602, Abcam, 1:1000 dilution) for 1 h at room temperature with gentle shaking. Membranes were incubated with a horseradish peroxidase - conjugated secondary antibody (1:5000 dilution) for 1 h under room temperature. The proteins were detected using a chemiluminescent reagent (Millipore), and the images were performed by GeneGnome XRQ-chemiluminescence image system (SYNGENE).

### Assessment of Trabecular Bone Parameters

The entire legs of mice with different age, gender and *Dnmt3l* genotypes were collected. After carefully removing the skin and most of the muscle, the intact femur, tibia, fibula with intact patella and joint were stored in 70% EtOH, and subject to 3D micro-CT imaging. The area of distal femur 0.4 to 1.9 mm from growth plate were used to analyze bone density and other trabecular parameters, with source voltage of 70 kV, source current of 57 μA, exposure for 1669 ms with AI 0.5 mm filter and image pixel size of 9 μm. Among the parameters, the elevated trabecular separation, reduced trabecular number, connectivity density and percent bone volume are associated with vertebral fracture (Qiu et al., 2006; Ostertag et al., 2009). On the other hand, increased SMI, decreased trabecular thickness and bone surface density are linked to with aging associated decline (Stauber and Muller, 2006; Chen et al., 2013).

## Data Availability Statement

The datasets presented in this study can be found in online repositories. The names of the repository/repositories and accession number(s) can be found below: https://www.ncbi.nlm.nih.gov/geo/query/acc.cgi?acc=GSE113844.

## Ethics Statement

The animal study was reviewed and approved by National Taiwan University IACUC Committee.

## Author Contributions

S-PL, FH, Y-PY, and P-YC conceived andplanned the experiments and analysis. C-YY, M-KL, FH, and Y-PY isolated and characterized MSCs from *Dnmt3l* mutant mice and wild type littermates. FH and Y-PY discovered the defected self-renewal and osteogenic differentiation potential from Dnmt3l KO epiphysis derived MSCs (EMSCs). P-SH and M-KL examined the *in vivo* bone density and various properties. Y-PY tested the hypothesis of DNMT3L legacy modulating protein expression of other epigenomic modulators in EMSCs. M-KL and C-YY discovered the phenotype of Dnmt3l KO BM-MSCS. C-CC performed bone fracture repair experiments. M-KL tested the unusual H3k9me3/H3K4me3 hypothesis in adipogenesis being one of the causes for preferred adipogenesis over osteogenesis in cultured *Dnmt3l^–/–^* MSCs. M-KL prepared MSC derived RNA for RL to perform RNA-sequencing library construction and bioinformatic analysis under P-YC’s supervision. C-YY and RL tested the ESC expressing DNMT3L mediated epigenetic legacy hypothesis. S-KC prepared the aging accelerating high fat diet and jet-lag paradigm model for the paired Dnmt3l KO and littermates, as well as associated analyses. C-YY, RL, M-KL, Y-TT, P-YC, and S-PL wrote the manuscript. All authors were involved in periodic debates and discussions towards development of the story and proved the final manuscript.

## Conflict of Interest

The authors declare that the research was conducted in the absence of any commercial or financial relationships that could be construed as a potential conflict of interest.

## Abbreviations

ARS: alizarin red S
BM-MSCs: bone marrow-derived MSCs
CFUs: colony forming units
CFU-F: colony forming unit-fibroblasts
DEGs: differentially expressed genes
DETEs: differentially expressed TEs
DNMT3L: DNA methyltransferase 3-like
ECM: extracellular matrix
EDTA: ethylenediaminetetraacetic acid
EMSCs: epiphysis-derived mesenchymal stem/stromal cells
ES: embryonic stem
FBS: fetal bovine serum
FDR: false discovery rate
HDAC1: histone deacetylase 1
IACUC: Institutional Animal Care and Use Committee
KD: knockdown
KO: knockout
MACS: magnetic-activated cell sorting
MEFs: mouse embryonic fibroblasts
α MEM: modified Eagle’s medium alpha
MOST: Ministry of Science and Technology
MSCs: mesenchymal stromal cells
OSF-2: osteoblast-specific factor-2
PCA: principal component analysis
PDT: population doubling time
PHD: plant homeodomain
POSTN: periostin
RIN: RNA integrity number
ROI: region of interest
SMI: structure model index
TEs: transposable elements
TSS: transcriptional start site
WGCNA: weighted gene co-expression network analysis
WT: wild-type.
