# Supplementary material for: Transcriptome Analysis of Dnmt3l Knock-Out Mice Derived Multipotent Mesenchymal Stem/Stromal Cells During Osteogenic Differentiation
Source: Front Cell Dev Biol. 2021 Feb 25;9:615098. doi: 10.3389/fcell.2021.615098 (PMC7947861; doi:10.3389/fcell.2021.615098)
Supplement: Supplementary file 2 [file Image_1.pdf]

Supporting Information

FIGURE S1

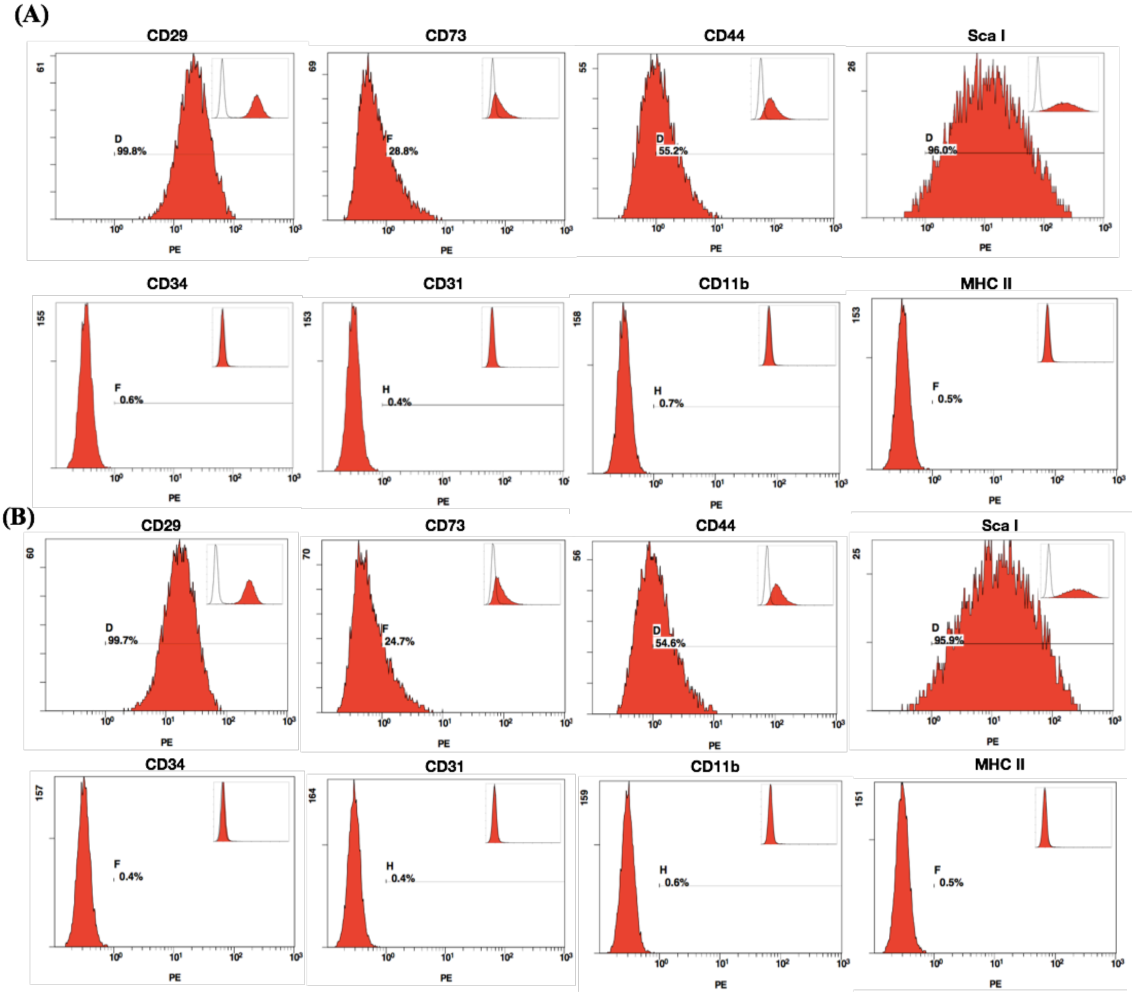

Figure S1. Surface markers expression profile analysis of MSCs by flow cytometry

Flow cytometric analysis was performed for MSCs at passage 2, derived from 15-16 weeks of *Dnmt3l* KO mice and wild type littermates (3 mice of each genotype), respectively. As expected, **(A)** wild type and **(B)** *Dnmt3l* KO MSCs were positive for MSCs markers (CD29, CD73, CD44 and ScaI), but negative for hematopoietic lineages markers (CD34 and CD11b), endothelial markers (CD31) and also showed low expression of MHC II. The black line indicates the respective isotype control.

Supporting Information

FIGURE S2

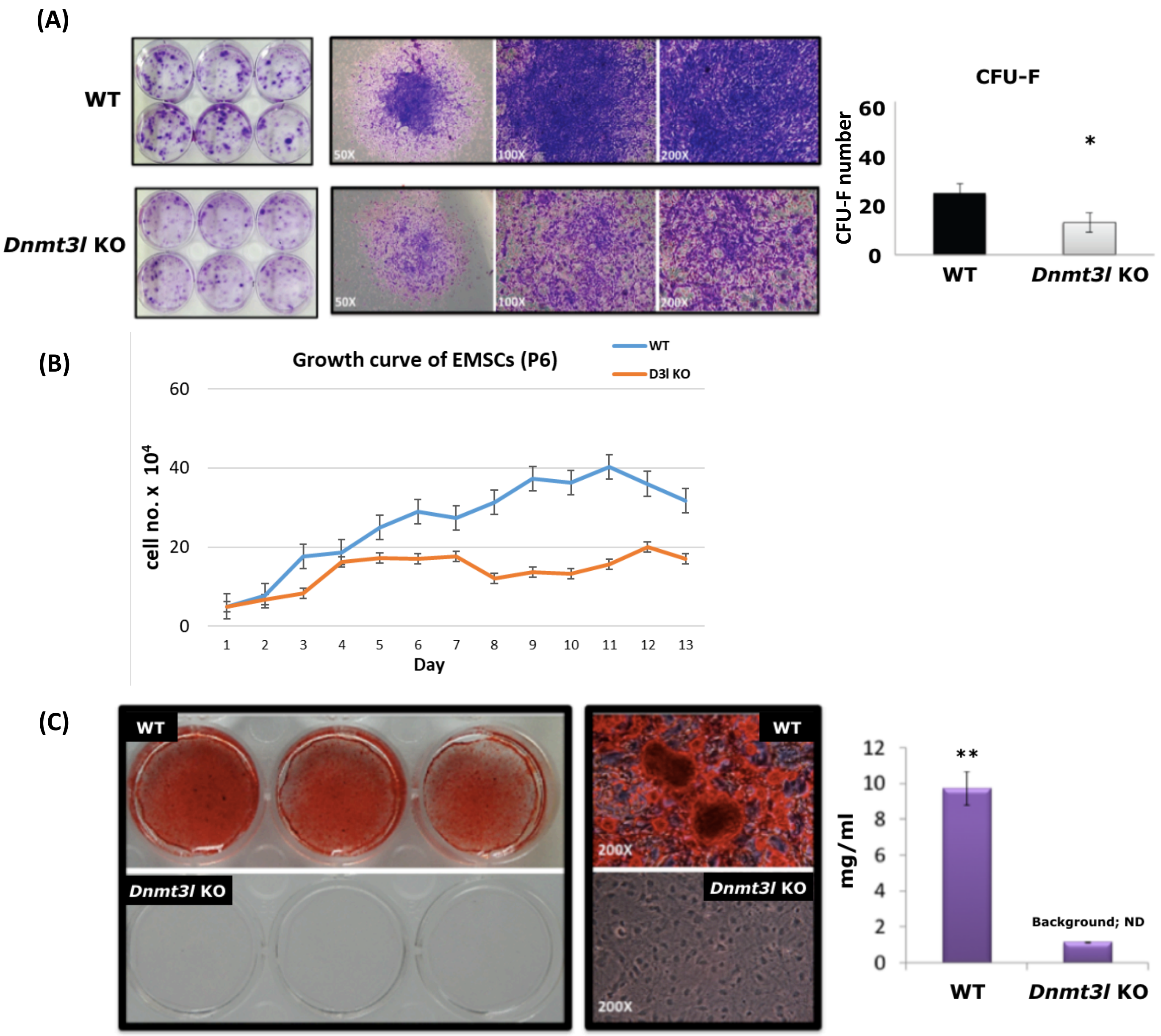

Figure S2. *Dnmt3l* deficiency impeded MSCs self-renewal potential and osteogenesis ability in an epiphysis-derived MSC (EMSCs) subtype

(A) The lack of *Dnmt3l* also impaired CFU-F forming efficiency in the EMSCs subtype at passage 4 (The cells were seeded at a density of 1000 cells per cm^2 in 6 well). (B) Reduced proliferation activity was observed in *Dnmt3l* KO mice derived EMSCs. (C) Osteogenesis of EMSC from WT and *Dnmt3l* KO mice were determined by Alizarin Red S staining at 7 days of osteogenic induction. Data presented as mean ± SEM. * $p < 0.05$, ** $p < 0.01$. A representative batch of experiment was presented. Independent experiments of MSCs from different batches of mice gave similar results.

Supporting Information

FIGURE S3

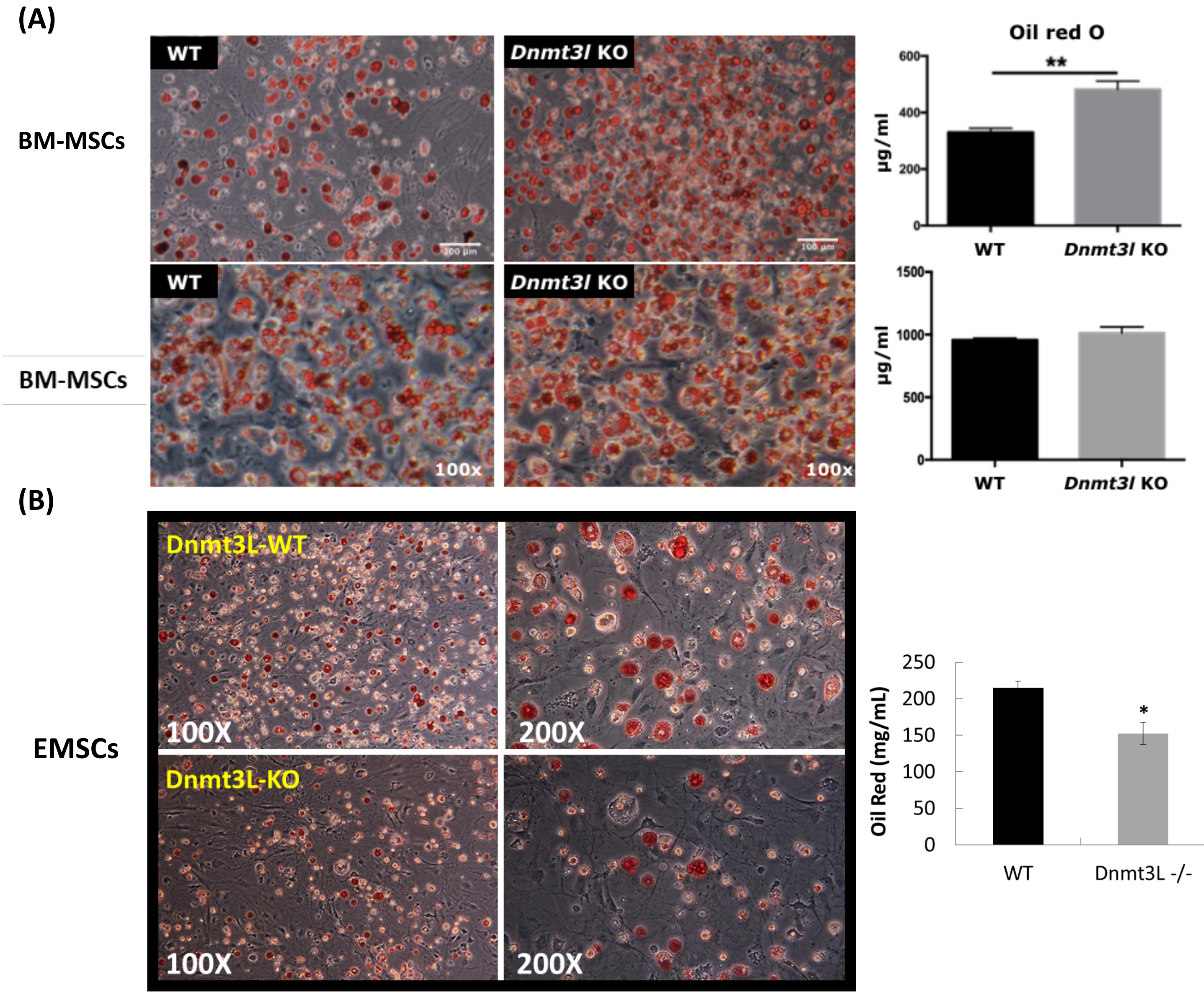

Figure S3. Adipogenesis activity observed from *Dnmt3l* KO BM-MSCs and EMSCs.
(A) Adipogenic capability was characterized by oil red O staining after 7 days of induction at passage 4. For quantification, oil red O-stained cells were solubilized at DMSO and the absorbance was measured spectrophotometrically at 570 nm. BM-MSCs derived from *Dnmt3l* KO mice had either equivalent or higher adipogenic potential compared to WT from different batches. (B) *Dnmt3l* KO EMSCs, on the other hand, have 30% reduced adipogenesis activity. Compared to the profound impairment on calcium deposition after osteogenic induction, the *Dnmt3l* genotype effect on adiopogenesis was relatively mild and subject to batch and MSC origin effect. Data presented as mean ± SEM. * $p < 0.05$, ** $p < 0.01$.

Supporting Information

FIGURE S4

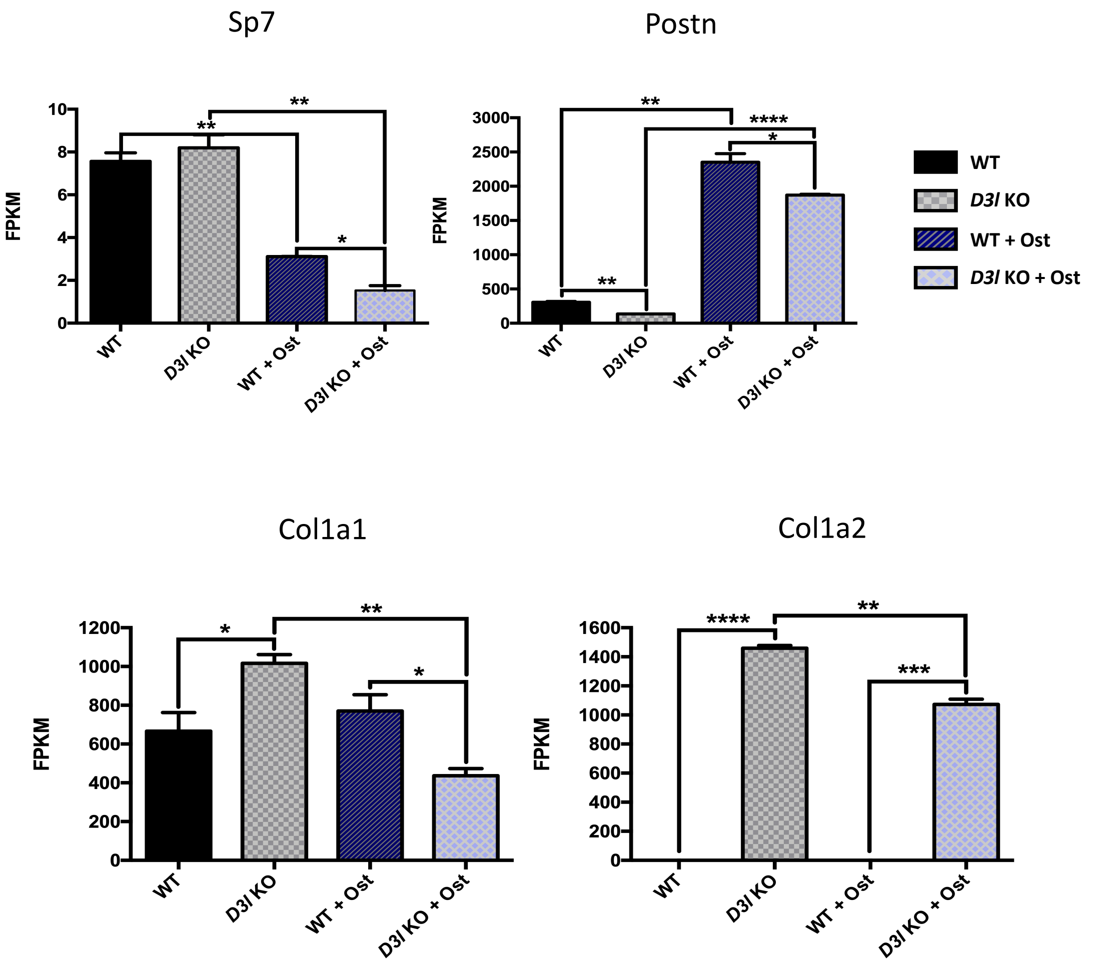

Figure S4. Analysis of candidate osteogenesis related gene expression in WT and *Dnmt3l* KO mice-derived MSCs before and during *in vitro* osteogenesis

Relative expression levels of candidate genes normalized by fragments per kilobase per million consolidated from our strand-specific transcriptome sequence datasets. Two-tail, unpaired *t*-test were used in all statistical analysis in this figure. *D3l KO and* WT represent undifferentiated passage 4 MSCs initially isolated from homozygous *Dnmt3l KO* animals or their wild type littermate; +Ost represents differentiating MSCs 3 days after osteogenesis induction.

Supporting Information

FIGURE S5

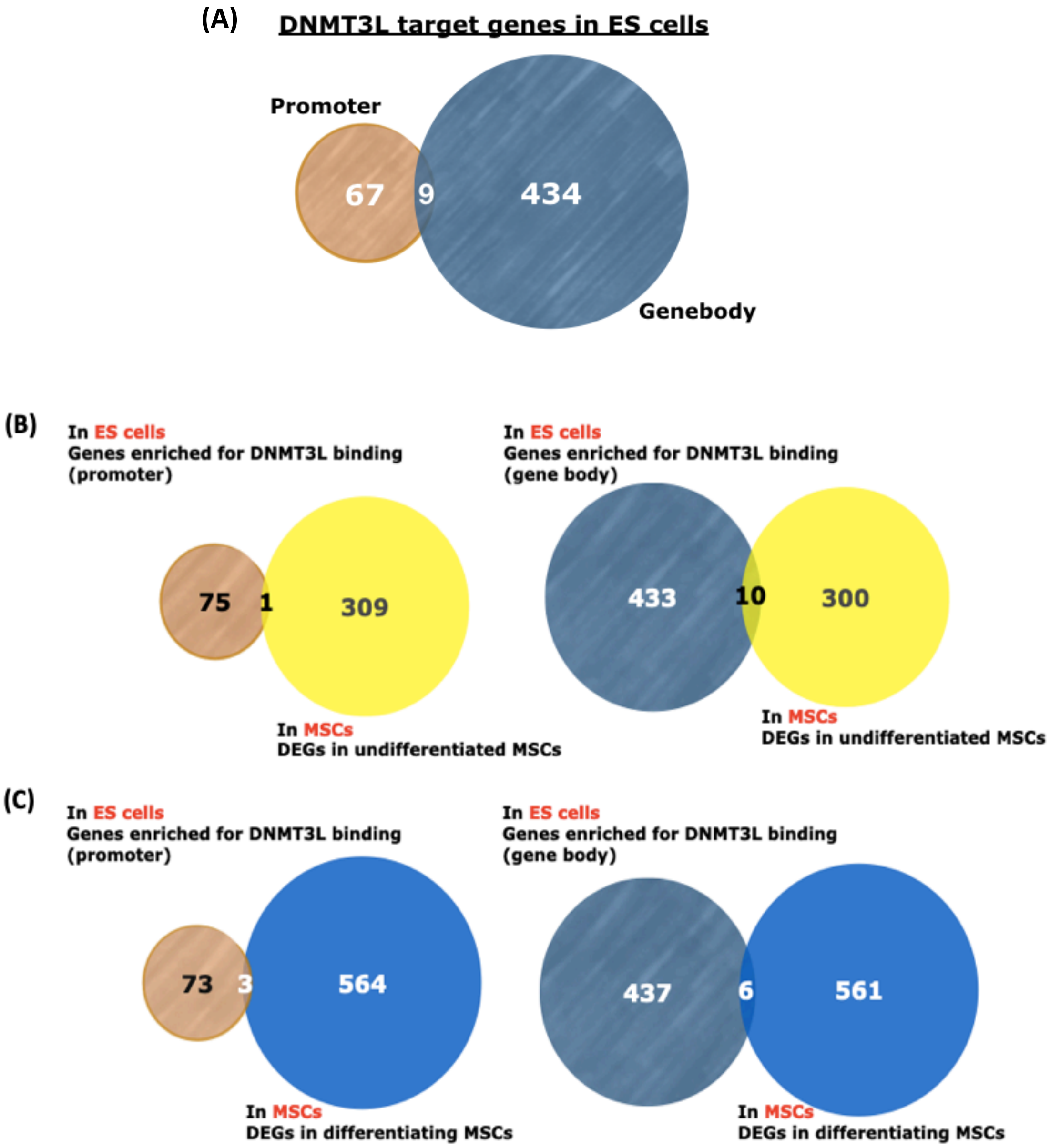

Figure S5. DNMT3L binding sites in ES cells and the limited correlation with the *Dnmt3l* genotype-dependent DEGs in undifferentiated and differentiating MSCs

(A) The number of DNMT3L targeted genes on promoter and gene body regions. (The 5 kb interval upstream of TSS were considered as putative promoter region) (B) Only 1 out of 308 DEGs between undifferentiated MSCs derived from WT and *Dnmt3l* KO mice are direct DNMT3L binding targets at promoter in ES cells (Left panel; p=0.616). Ten out of 308 DEGs between undifferentiated MSCs derived from WT and *Dnmt3l* KO mice are DNMT3L binding targets at gene body in ES cells (Right panel; p=0.0584). (C) Three out of 567 DEGs between differentiating MSCs derived from WT and *Dnmt3l* KO mice are DNMT3L binding targets at promoter in ES cells (Left panel; p=0.260). Six out of 567 DEGs between differentiating MSCs derived from WT and *Dnmt3l* KO mice are DNMT3L binding targets at gene body in ES cells (Right panel; p=0.984). P values were calculated by hypergeometric test.

Supporting Information

FIGURE S6

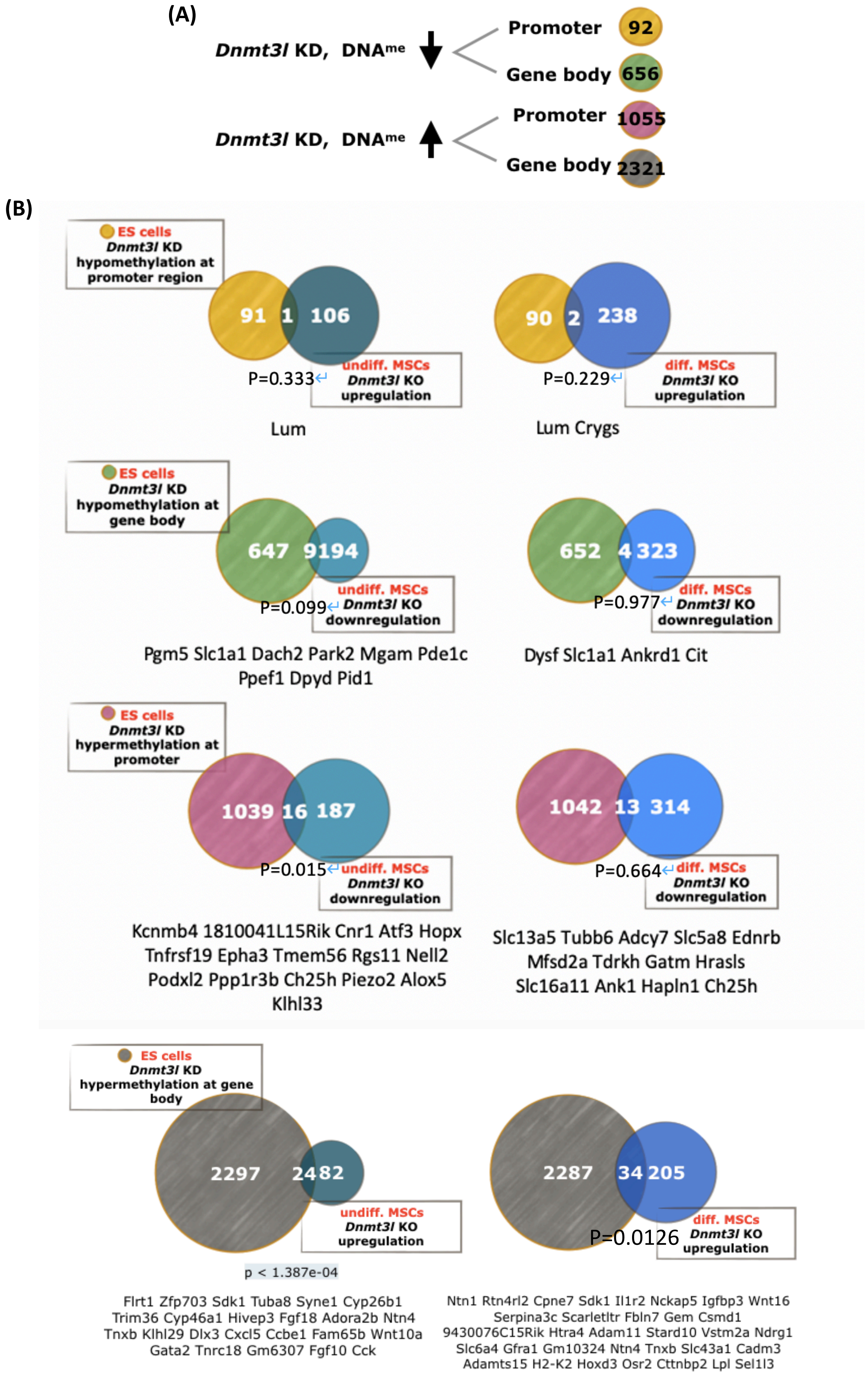

Figure S6. The relatively higher proportion of DEGs between *Dnmt3l* KO and WT MSCs are overlapped with genes bearing DNMT3L-associated gene body DNA methylation in ES cells

(A) Among the 748 genes hypomethylated in *Dnmt3l* KD ES cells compared to WT counterparts, 92 genes are hypomethylated at putative promoter regions and the other 656 genes at gene body regions. In contrast, 3376 genes are hypermethylated in *Dnmt3l* KD ES cells compared with WT, of which 1055 genes are hypermethylated at putative promoter regions and the other 2321 genes at gene body regions. We define the 5kb upstream of TSSs as putative promoter regions. (B) DNA methylation on the promoter usually resulted in downregulation of gene expression. We therefore compared the 92 genes (yellow circle) with hypermethylated promoter in wild type ES cells, with the up-regulated genes in undifferentiated and differentiating *Dnmt3l* KO MSCs. Only 1 and 2 genes were overlapped, respectively, indicating DNA methylation on the putative promoter regions in the ES cells, were less likely to be carried over to MSCs to affect gene expression. On the other hand, 1055 genes (pink circle) with promoter hypermethylation in *Dnmt3l* KD ES cells were compared with the down-regulated genes in undifferentiated and differentiating *Dnmt3l* KO MSCs. Since DNA methylation on gene body generally resulted in upregulation of gene expression, the 656 genes (green circle) with gene body hypomethylation in *Dnmt3l* KD ES cells were compared with the down-regulated genes in undifferentiated and differentiating *Dnmt3l* KO MSCs. The 2321 genes (gray circle) with gene body hypermethylation in *Dnmt3l* KD ES cells are compared with the up-regulated genes in undifferentiated and differentiating *Dnmt3l* KO MSCs. Higher proportion (24/106) of *Dnmt3l* genotype dependent differentially expressed genes in undifferentiated MSCs correlated to genes with gene body methylation in embryonic stem cells. P values were calculated by hypergeometric test.

Supporting Information

FIGURE S7

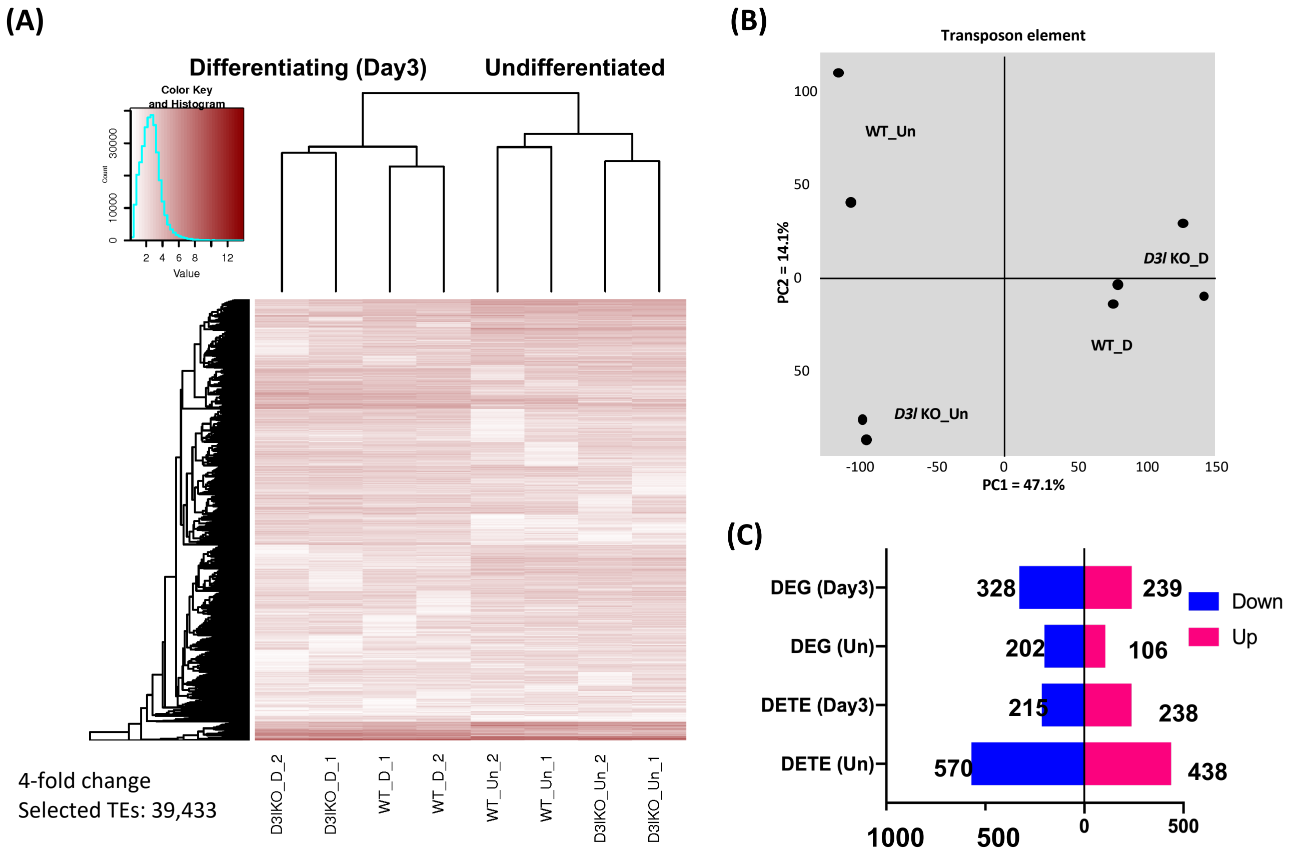

Figure S7. Genotype and differentiation mediated differential expression of transposable elements

(A) Global transposon element expression in undifferentiated and differentiating (day 3 of osteogenic induction) MSCs derived from WT and *Dnmt3l* KO mice. (B) Principal Component Analysis (PCA) of transposon element expression. (C) The number of DEGs and DETEs between WT and *Dnmt3l* KO in undifferentiated and osteogenic differentiating MSCs.

Supporting Information

FIGURE S8

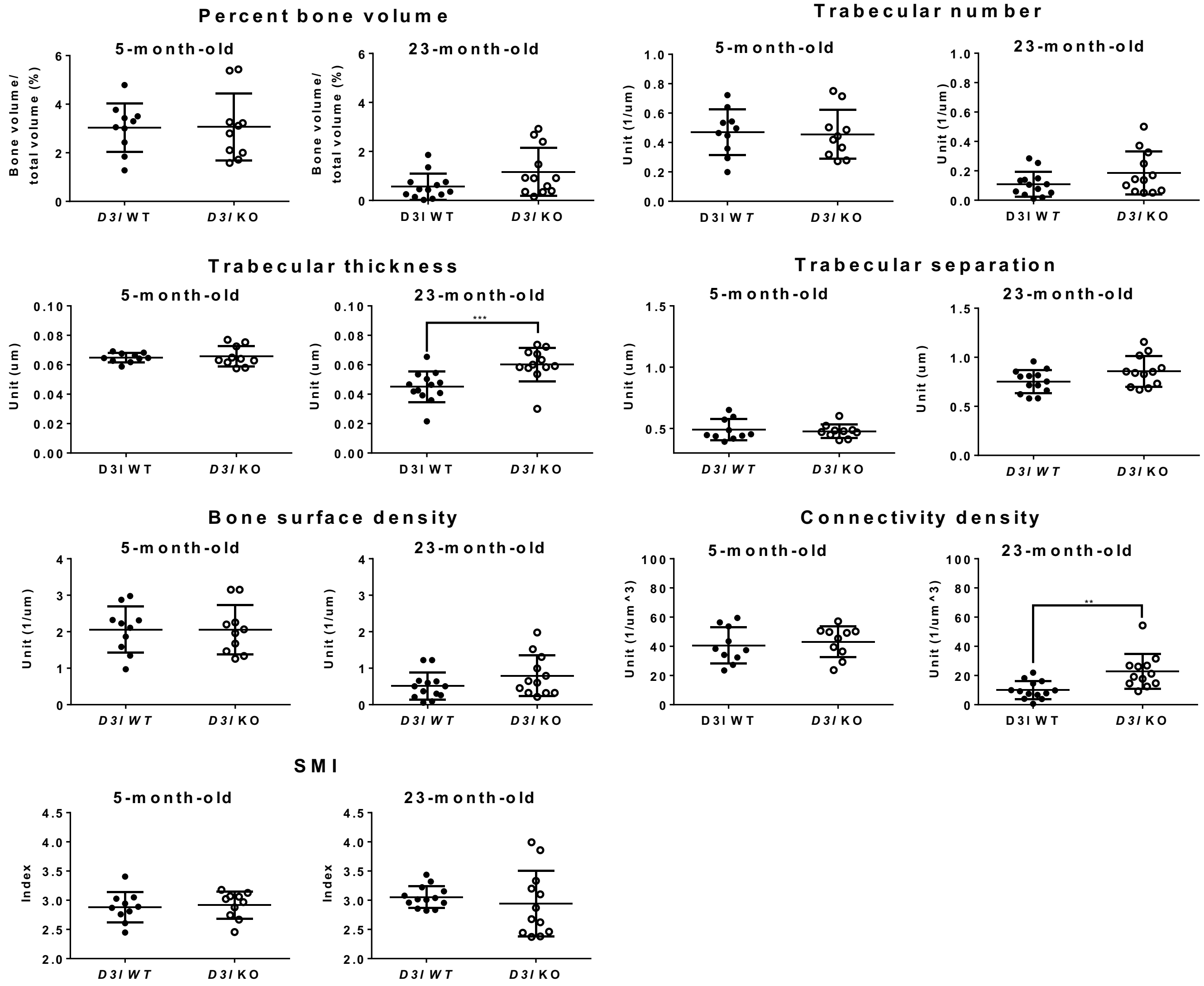

Figure S8. Trabecular parameters analyzed from 5-month- and 23-month-old paired *Dnmt3l* female KO mice and wild type littermates

Bone volume, trabecular numbers, trabecular thickness, Trabecular separation, bone surface density, connectivity density and structure model index (SMI) were compared between (A) 5-month-old and 23-month-old WT and *Dnmt3l* KO female mice. *Dnmt3l* KO genotype surprisingly have protective effect against aging associated connectivity density and trabecular thickness reduction phenotype. Statistic differences were analyzed by Mann-Whitney test. P<0.05*, <0.01**, <0.001*** and <0.0001****.

Supporting Information

FIGURE S9

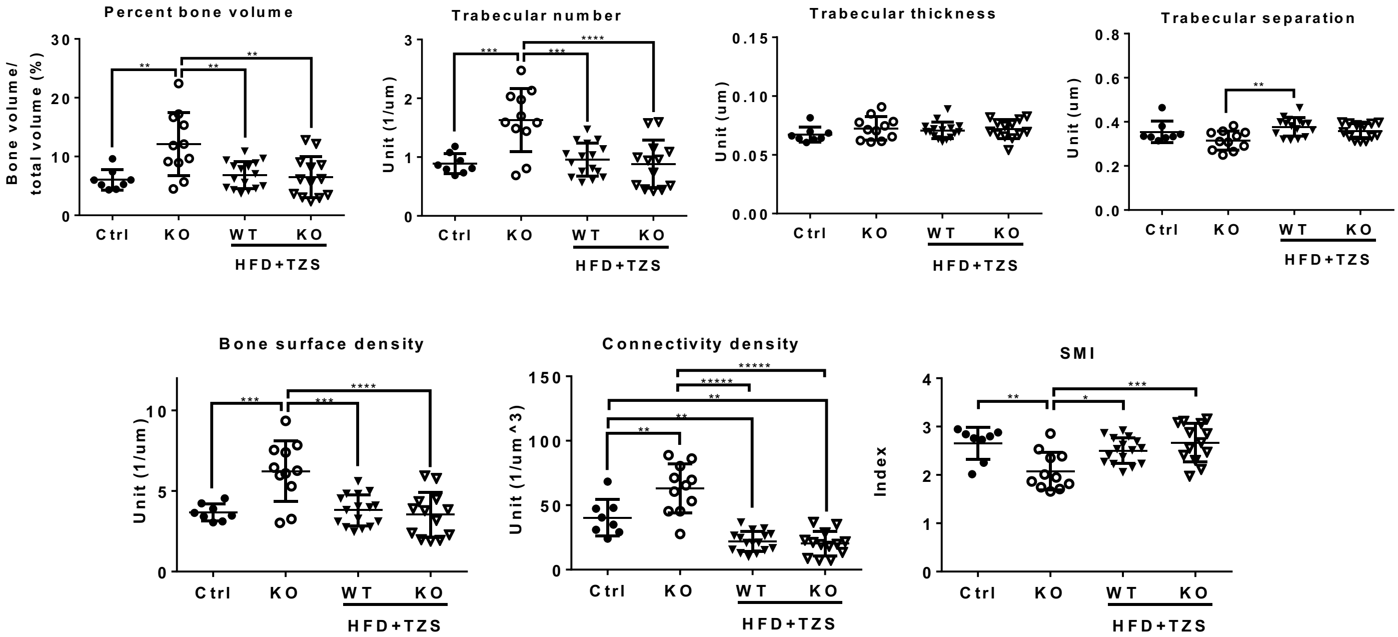

Figure S9. *Dnmt3l* KO male mice demonstrated better bone quality compared with heterogeneous *Dnmt3l+/-* and WT male littermates but sensitized to high fat diet plus an experimental jet-lag paradigm/Time zone shifting (H+T).

Bone volume, trabecular numbers, trabecular thickness, Trabecular separation, bone surface density, connectivity density and structure model index (SMI) were compared between 7 to 8-months-old *Dnmt3l* WT/heterogeneous and *Dnmt3l* KO male mice with breeding diet (untreated) or with "H+T": high fat diet (#D12492, Research diets) plus an experimental jet-lag paradigm: the 6-months-old experimental mice were fed with high fat diet accompanied with light/dark cycle shifting forward for 6 hours/week until the mice became 8-month-old. Control WT and *Dnmt3l* KO mice were kept in normal light/dark cycle and fed with breeding diet. Statistic differences were analyzed by one-way ANOVA with Tukey's test. P<0.05*, <0.01**, <0.001*** and <0.0001****.

Table S1. Summary of RNA-Seq data of osteolineage differentiating and undifferentiated BM-MSCs derived from WT and *Dnmt3l* KO mice

Samples	Samples description	Raw reads	Mapped reads	Mappability (%)
WT_Un_1	WT, undifferentiated MSCs	65,896,914	63,480,135	96.3
WT_Un_2		67,218,537	65,137,093	96.9
WT_D_1	WT MSCs on day 3 of differentiation towards osteoblasts	90,747,616	86,985,776	95.9
WT_D_2		68,753,361	66,094,575	96.1
Dnmt3l KO_Un_1	Dnmt3l KO derived undifferentiated MSCs	65,743,245	62,254,628	94.7
Dnmt3l KO_Un_2		72,705,565	66,035,878	90.8
Dnmt3l KO_D_1	Dnmt3l KO derived MSCs on day 3 of differentiation towards osteoblasts	66,684,124	64,244,082	96.3
Dnmt3l KO_D_2		67,805,802	64,496,488	95.1

*Read length = 51 bp

Table S2. Number of differentially expressed transposons in primary subfamilies

Family	Total number	Undifferentiated		Differentiating	
		up	down	up	down
**DNA**					
hAT-Charlie	105,698	10	20	9	3
TcMar-Tigger	35,118	2	8	2	3
hAT-Tip100	9,436	1	0	2	1
**LINE**					
L1	905,228	135	60	32	15
L2	67,909	7	18	11	3
CR1	14,155	2	2	4	1
**SINE**					
Alu	574,557	56	97	30	36
B4	397,726	33	37	14	87
B2	372,923	37	53	28	10
MIR	120,436	13	21	16	6
**LTR**					
ERVL-MaLR	454,918	66	67	19	17
ERVK	323,502	34	132	50	14
ERVL	118,581	15	10	6	3
ERV1	72,095	15	4	6	3
total		426	529	229	202
